# The Rhizosphere Bacterial Microbiota of *Vitis vinifera* cv. Pinot Noir in an Integrated Pest Management Vineyard

**DOI:** 10.3389/fmicb.2017.01528

**Published:** 2017-08-14

**Authors:** Giorgia Novello, Elisa Gamalero, Elisa Bona, Lara Boatti, Flavio Mignone, Nadia Massa, Patrizia Cesaro, Guido Lingua, Graziella Berta

**Affiliations:** ^1^Dipartimento di Scienze e Innovazione Tecnologica, Università degli Studi del Piemonte Orientale Alessandria, Italy; ^2^SmartSeq s.r.l. Alessandria, Italy

**Keywords:** microbiota, rhizosphere, grapevine, metagenome, phenological stages

## Abstract

Microorganisms associated with *Vitis vinifera* (grapevine) can affect its growth, health and grape quality. The aim of this study was to unravel the biodiversity of the bacterial rhizosphere microbiota of grapevine in an integrated pest management vineyard located in Piedmont, Italy. Comparison between the microbial community structure in the bulk and rhizosphere soil (variable: space) were performed. Moreover, the possible shifts of the bulk and rhizosphere soil microbiota according to two phenological stages such as flowering and early fruit development (variable: time) were characterized. The grapevine microbiota was identified using metagenomics and next-generation sequencing. Biodiversity was higher in the rhizosphere than in the bulk soil, independent of the phenological stage. Actinobacteria were the dominant class with frequencies ≥ 50% in all the soil samples, followed by Proteobacteria, Gemmatimonadetes, and Bacteroidetes. While Actinobacteria and Proteobacteria are well-known as being dominant in soil, this is the first time the presence of Gemmatimonadetes has been observed in vineyard soils. *Gaiella* was the dominant genus of Actinobacteria in all the samples. Finally, the microbiota associated with grapevine differed from the bulk soil microbiota and these variations were independent of the phenological stage of the plant.

## Introduction

*Vitis vinifera* (grapevine) is a typical Mediterranean crop with a very relevant impact on the Italian landscape, economy and culture. In 2016 more than 687.000 ha of the agricultural land in Italy was cultivated with grapevine, leading to a total yield of 84.000 tons of fruits (ISTAT, 2016)^1^.

In the Piedmont region of Italy, grapevine cultivation includes 44000 ha with a yield of 3.674 tons of high quality wine (about 6% of the total national production). Besides the economic importance, grapevine culture has an historical value in Piedmont; in June 2014 the hills of the area between Langhe, Roero and Monferrato have been declared a “Unesco World Heritage.”^2^

Improving the knowledge of a vine ecosystem can contribute to the characterization of the “terroir,” previously defined as “an interactive ecosystem, in a given place, including climate, soil and the vine (cultivar and rootstock)” (Seguin, 1988). This reflects the fact a wine produced in a given region is unique and cannot be reproduced elsewhere, even if the grape cultivar and winemaking procedures are the same. Typical wine features depend on the fruit composition which is a consequence of growth in a specific geographical region, specific chemical and physical soil parameters, climate and by the specific interactions between the plant and the biotic and abiotic components of the surrounding environment. Therefore, the terroir may also be influenced by the local soil microbiota, including microorganisms living both surrounding and inside plant tissues, that in turn can potentially affect grapevine health and wine quality. Plant beneficial microorganisms inoculated at the root level can modulate the fruit or edible seed composition, therefore affecting their nutritional value organoleptic quality (Giovannetti et al., 2012; Lingua et al., 2013; Berta et al., 2014; Bona et al., 2015, 2016; Flores-Felix et al., 2015), contributing to both yield and quality. On the other hand, the release of root exudates selects specific populations thereby affecting the structure of the microbial communities. As the composition and amount of rhizodeposit changes during a plant’s life, the microbial communities change concomitantly (Bulgarelli et al., 2013).

In the past 10 years, the soil microbiome associated to *V. vinifera* has received considerable attention. Among the microorganisms living in the rhizosphere, arbuscular mycorrhizal fungi (AMF) not only improve *V. vinifera* growth, both under natural and stressed conditions (for an excellent review see Trouvelot et al., 2015), but also affect its proteome leading to changes on berry quality, occurring especially on phenolic molecules synthesis (Cangahuala-Inocente et al., 2011). The biodiversity of AMF colonizing *V. vinifera* has been described by Holland et al. (2014), who recorded over 40 different taxa associated to vines mainly ascribed to the *Funneliformis* and *Rhizophagus* genera.

Very recently, the impact of both a single AM fungus and a mixed bacterial and fungal commercial inoculum on the transcriptome of Pinot Noir plants has been characterized by Balestrini et al. (2017); the data obtained underlined the presence of several genes upregulated by the two inocula, mainly involved in nitrogen metabolism, thus suggesting that these beneficial microroganisms are involved in stimulating plant responses to this element which is essential for grapevine metabolism.

Regarding bacterial population associated to grapevine, both epiphyte and endophyte bacterial communities have been investigated in a number of studies. For example, the identification of culturable endophytic bacteria from inside grapevine tissues, as well as the isolation and characterization of rhizospheric bacteria was reported by Karagöz et al. (2012) and Baldan et al. (2014). However, it has been estimated that only 1–10% of soil bacteria are culturable (Hugenholtz and Pace, 1996), and the percentage of culturable bacteria in rhizosphere change according to the host plant (Schlaeppi and Bulgarelli, 2015). Therefore, molecular tools are essential in order to gain a deeper knowledge of the diversity of grapevine bacterial communities. Different molecular procedures have been then used in order to gain information on the microbiota of *V. vinifera*. Bacterial endophytes living inside grapevine tissue were previously described by West et al. (2010) and Bulgari et al. (2014), while the analysis of epiphyte bacteria of fruits, leaves, and bark was performed by Martins et al. (2013). Furthermore, the impact of organic or conventional management on the grapevine rhizobacteria was described by Vega-Avila et al. (2015). In addition, the variability of the leaf Eukaryotic and Prokaryotic epiphytic community was characterized according to the plant developmental stage (Pinto et al., 2014).

Recent advances in next-generation sequencing (NGS) strategies can effectively help to disentangle complex microbial communities in specific ecological niches such as grapevine. Pyrosequencing techniques have revolutionized genomics and metagenomics and can generate sequence data for 100s of 1000s of DNA fragments from both culturable and non-culturable microorganisms, therefore providing a huge amount of information regarding the contents of a specific microbiome (Oulas et al., 2015).

Recently, by using this molecular approach, the diversity of bacterial communities was assessed in the most commonly grown grape cultivars in California (Bokulich et al., 2014). Then, the shifts of the microbial communities inside plant tissue as a consequence of infection by flavescence dorée phytoplasma (Bulgari et al., 2014) and the management of the vineyard (Campisano et al., 2014a) were described. Finally, insight on the variability of grapevine microbial community structure of leaves, flowers, grapes, roots and soil during three phenological stages of grapevine was provided Zarraonaindia et al. (2015). However, no one of these papers focused on the shifts of the grapevine microbiota according to both the plant presence and age. This paper aims to fill a real gap in the knowledge of the dynamic of the microbial communities associated to grapevine. Therefore, we characterized, by a metagenomic approach, the microbiota of the roots of *V. vinifera* cv. Pinot Noir, in a vineyard subjected to integrated pest management (IPM), paying special attention to the shifts induced by (i) the phenological stage through the comparison between flowering and early fruiting time; this period encompass the opening of the flower and the development of fruit, which are very different phases of plant development under a physiological, metabolic, and hormonal points of view and (ii) the rhizosphere effects through the comparison between bulk and rhizosphere soil.

## Materials and Methods

### Soil Sampling

The IPM vineyard is located close to Carpeneto (AL) Altitude: 286 m a.s.l., Latitude: 44,683706°N and Longitude: 8,6258889°E. In Europe, IPM is not yet regulated; however, its general principles are listed in the Annex III of Directive 2009/128/EC. According to this directive, (i) soil borne disease suppression and prevention should be based on crop rotation, use of resistant cultivar and adequate fertilization and irrigation; (ii) sustainable biological methods are preferred to pesticide for the control of plant pathogens; (iii) if the use of pesticide is necessary the most selective and the less dangerous for organisms and environment should be chosen and distributed in low amount and with low frequency. IPM aims to grow healthy crops with the least possible disruption to agroecosystems and encourages natural pest control mechanisms (Matyjaszczyk, 2015).

Data regarding the temperature, the humidity and rainfall are reported in Supplementary Figure 1.

The soil is clay loam (USDA, sand 29.8% silt 41.3% clay 28.9%), slightly alkaline (pH 7.89), with a total organic carbon 4.3 g/Kg, total Nitrogen 0.69 g/Kg, C/N 6.30, and cation exchange capacity CEC 15.9 meq/Kg.

Chemical treatments performed during vine growth were weeding with glyphosate (in April) among the plants, but not between the lines, fungicide treatment (Metalaxil-m + mancozeb) against *Peronospora* spp. and (Ciflufenamid) *Oidium* spp. each month from April to the end of fruitification, fungicide treatment (Cyprodinil + Fludioxonil) against *Botrytis cinerea* in July, and two insecticide (Thiamethoxam + Chlorpyrifos-metile) treatments in July.

Soil samplings were performed in May and July 2014, corresponding to flowering and early fruit development, respectively. The bulk soil (BS1 and BS2, for each sampling date) and the soil associated with the roots of *V. vinifera* cv. Pinot noir (Rhiz1 and Rhiz2, for each sampling date), five per each kind, were sampled at a depth of 30 cm, corresponding to the topsoil, after removing the surface layer (3.0–5.0 cm). Three soil cores were taken in the proximity of the stem (3 cm), therefore a total of 15 cores were taken for each plant phenological stage. The roots entrapped in the soil cores collected close to the stem were considered for the sampling of rhizosphere soil. The soil adhering to these roots was removed using sterile gloves. As recommended by the Italian law (GU 179/2002) for soil characterization analysis, the three subsamples of rhizosphere and bulk soil were then pooled in order to obtain a homogeneous sample.

Soil samples were then stored at -20°C for 1 week for DNA extraction.

### DNA Extraction

DNA was extracted directly from 0.25 g of soil using the Power Soil^®^ DNA Isolation Kit (MO BIO Laboratories, Inc., Carlsbad, CA, United States) following the manufacturer’s instructions. Extracted DNA was visualized following electrophoresis on an 0.8% agarose gel in 1xTAE buffer [40 mM Tris (pH 7.6) 20 mM acetic acid, 1 mM EDTA]. The DNA was then subjected to ethanol precipitation. DNA amount and purity were evaluated by spectrophotometric absorbance measurement at λ 260 nm, 260/280 nm and at λ 260/230 nm, respectively before and after the precipitation. A ratio λ 260/280 of 1.8 indicates pure DNA; expected values for the ratio λ 260/230 is 2.0–2.2.

### DNA Amplification and Roche 454 Pyrosequencing

DNA extracted from the five samples of bulk soil (BS) and rhizosphere (Rhiz) harvested during flowering and fruit development were amplified with primers for the V1 (5′-AGAGTTTGATCCTGGCTCAG-3′) (Weisburg et al., 1991) and V4 (5′-CTACCAGGGTATCTAATC-3′) (Wang and Qian, 2009) regions of 16S rDNA tagged with Multiplex Identifier sequences for 454 Pyrosequencing (Roche). The reaction was performed in a Techne thermocycler (TC512, Bibby Scientific, Riozzo di Cerro al Lambro, Italy) and the conditions including an initial denaturation at 94°C for 5 min; 34 cycles at 94°C for 1 min, 60°C for 1 min, and 72°C for 5 min; and a final elongation at 72°C for 10 min. Each reaction mixture (20 μl) contained 5 ng of soil DNA, 100 μM of dNTPs DNA, 1.5 mM MgCl_2_, 1× Buffer [67 mM Tris-HCl pH 8.8; 16.6 mM (NH_4_)_2_SO_4_; 0.01% Tween-20; MgCl_2_ 5 mM], and 0.08 U of Taq DNA Polymerase (Thermofisher) and DMSO 5%.

Polymerase chain reaction products were used for pyrosequencing with 454 technology; amplicons were amplified in droplet water in oil emulsions. DNA-carrying beads were loaded into individual wells on a PicoTiter^TM^ plate and surrounded by enzyme beads (sulfurylase luciferase). Nucleotides were flowed one at a time over the plate and template-dependent incorporation released pyrophosphate, which was converted to light through luciferin/luciferase enzymatic reaction. The light signals were represented in flow grams and analyzed; a nucleotide sequence was determined for each read with the GS Amplicon Variant Analyzer software.

### Bioinformatic and Statistical Analysis

Data were analyzed using a custom bioinformatics pipeline. Raw sequence reads were demultiplexed to obtain a single file for each sample (consisting of 5 biological replicates × 2 plant phenological stages × 2 soil sites classified as bulk and rhizosphere soil). During this process, reads that met the following criteria were discarded: (1) read length < than 200 nt, (2) average Phred quality score (Ewing et al., 1998) < than 25, (3) read contained at least one ambiguous base.

For each sample, the taxonomic assignment up to genus level was performed using RDP^3^ classifier (Wang et al., 2007) and species-level resolution was attained by blasting reads against a core set of the RDP database.

Sequences have been deposited in a specific BioProject (PRJNA394211) in GenBank; they were clustered according to similarity thresholds (≥97%) and the representative sequence of each cluster was identified with the name of the corresponding RDP hit for all taxonomic levels.

Finally, a table with absolute abundance for all soil samples was used as input for the analysis with RAM package of R statistical software 3.1.3^4^ to obtain: (1) the alpha diversity graphs, (2) PCA ordination, (3) the biodiversity indices (Shannon–Wiener Index, Simpson Index, Observed species).

Statistical analysis was performed with R statistical software. Data were compared by a non-parametric Mann–Whitney test with cut-off significance at *p* < 0.05 to assess differences between treatments.

## Results

**FIGURE 1:**
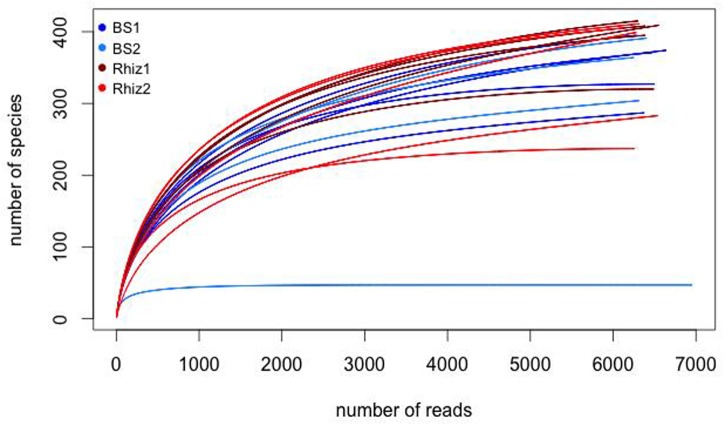
Rarefaction curves for each sample (BS1, BS2, Rhiz1, Rhiz2, five sub-samples each).

The comparison of prokaryotic diversity was performed analyzing the rarefaction curves (**Figure 1**). Rarefaction curves are based on the observation that the curve of rarefied counts of any feature should plateau if the sample is close to saturation (Rodriguez-R and Konstantinidis, 2014) thus providing a measure of the depth of our experiments. According to **Figure 1**, the number of observations was sufficient to obtain a good estimate of the species richness in our samples and an efficient coverage of the entire community was achieved. A total of 142908 reads were obtained with a mean value of 7500 reads per sample. Sequences were demultiplexed in order to split the input data into a single file for each sample and filtered based on standardized parameters such as minimum value of the sequence quality, minimum length nucleotide sequence and number of “mismatches” for sequences of primer and barcode. After this step, a total of 128296 reads (with a mean value of 6800 reads per sample) were used for further analysis. As seen in the rarefaction curves, the number of reads coming from one sample of bulk soil harvested during the time corresponding to the flowering stage of the plant was 10 times lower than the that of the other soil samples.

### Biodiversity

**FIGURE 2:**
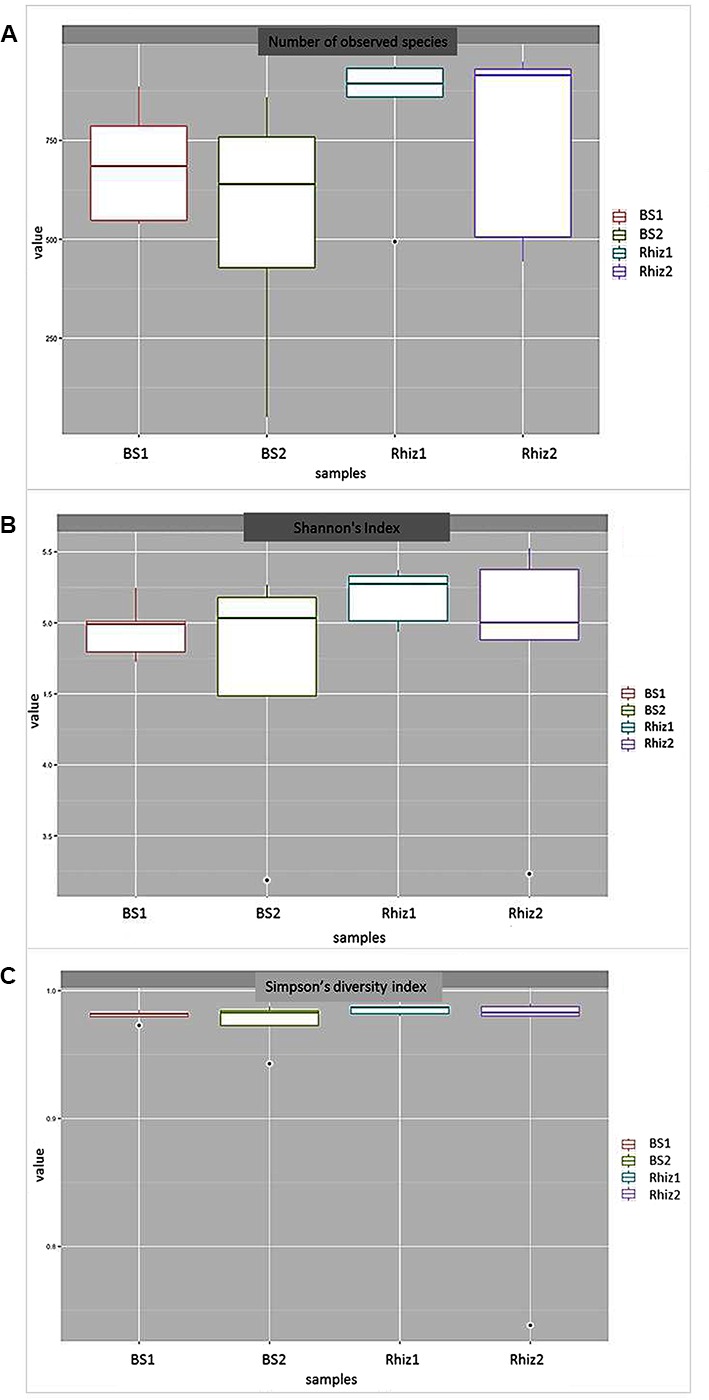
**(A)** Number of bacterial species detected in bulk soil and rhizosphere of *Vitis vinifera* at the two sampling times. **(B)** Biodiversity (Shannon’s Index) of the microbial community found in bulk soil and the rhizosphere at the two sampling times. **(C)** Simpson’s Diversity Index of the microbial community found in bulk soil and the rhizosphere at the two sampling times.

In order to measure alpha diversity (i.e., the local diversity of a community) the calculation of three estimators was performed. The median number of bacterial species was similar in the two samplings; however, this parameter was higher in the rhizosphere (first sampling, 894; second sampling, 915) than in the bulk soil (first sampling 685; second sampling 639.5) (**Figure 2A**). The median value of the Shannon–Wiener’s Index, that is an entropy measurement that increases according to the number of species in the sample, was higher in the rhizosphere at the first sampling (flowering) than in all the other cases (**Figure 2B**). The Simpson’s Index, which is based on the probability of assigning two independent individuals taken randomly from the community into the same species, did not change in the different samples (**Figure 2C**).

### Description of Microbial Communities

**FIGURE 3:**
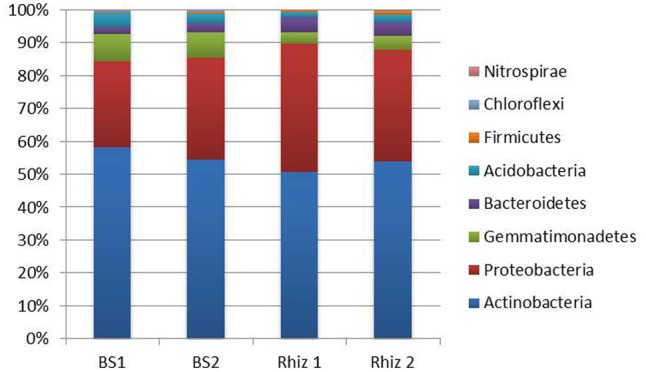
Microbial community composition in the bulk soil and rhizosphere of *V. vinifera* cv. Pinot Noir at the two sampling times (flowering and early fruiting) at the phylum level (top 8 taxa).

A total of 128296 reads were obtained for phyla description. Actinobacteria were the dominant phylum (**Figure 3**); their frequency (BS1 58.24%; BS2 54.60%; Rhiz1 50.65%; Rhiz2 53.85%) did not significantly change between the soil samples. Similarly, the number of reads ascribed to Proteobacteria did not change significantly as a function of sampling time or site (BS1 26.20%; BS2 30.81%; Rhiz1 39.00%; Rhiz2 33.95%). Abundance of Gemmatimonadetes and Chloroflexi differed significantly between bulk soil (8.15 and 0.45%, respectively) and rhizosphere (3.45 and 0.03%, respectively) at the first sampling (flowering) (*p* = 0.032 and *p* = 0.012, respectively). No significant differences were observed in the frequency of Bacteroidetes, Acidobacteria, and Firmicutes phyla as a function of sampling time or site (**Figure 3**).

The amount of Nitrospirae recorded in bulk soil showed significant variations with time (BS1 0.06%; BS2 0.01%; *p* = 0.018) and also space, but only at the second sampling (fruit development) (BS2 0.01%; Rhiz2 0.09%; *p* = 0.016) (**Figure 3**).

**FIGURE 4:**
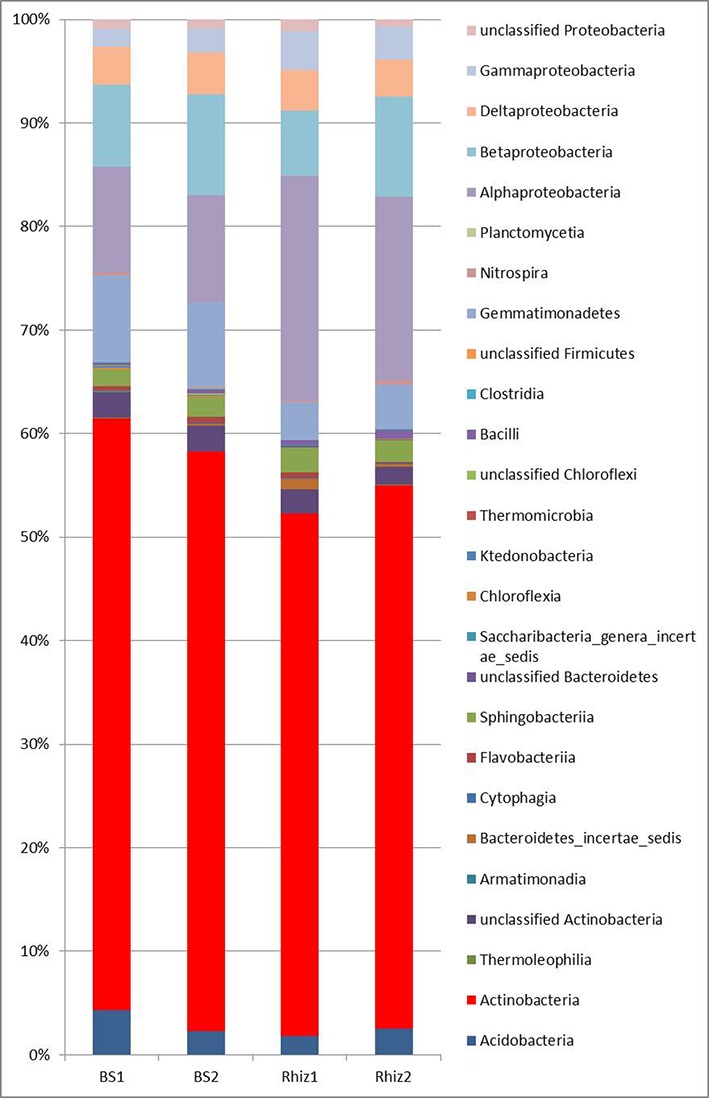
Microbial community composition in the bulk soil and rhizosphere of *V. vinifera* cv. Pinot Noir at the two sampling times (flowering and early fruiting) at the class level.

**FIGURE 5:**
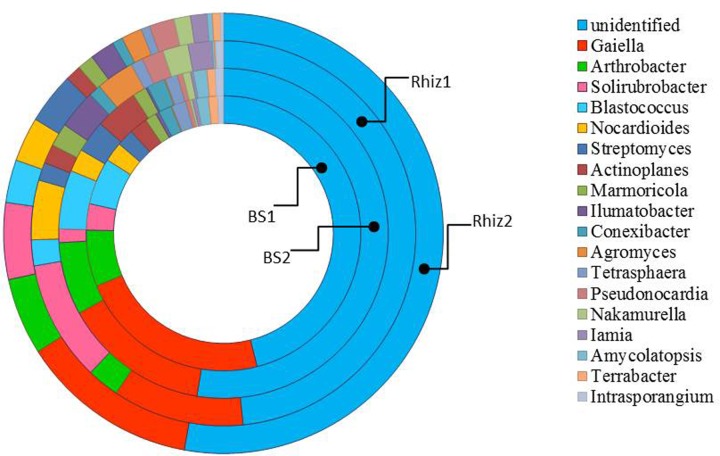
Distribution of the genera belonging to the class Actinobacteria in bulk soil and rhizosphere of *V. vinifera* cv. Pinot Noir during the two sampling dates (flowering and early fruiting). From the center to the edge BS1, BS2, Rhiz1, Rhiz2.

**FIGURE 6:**
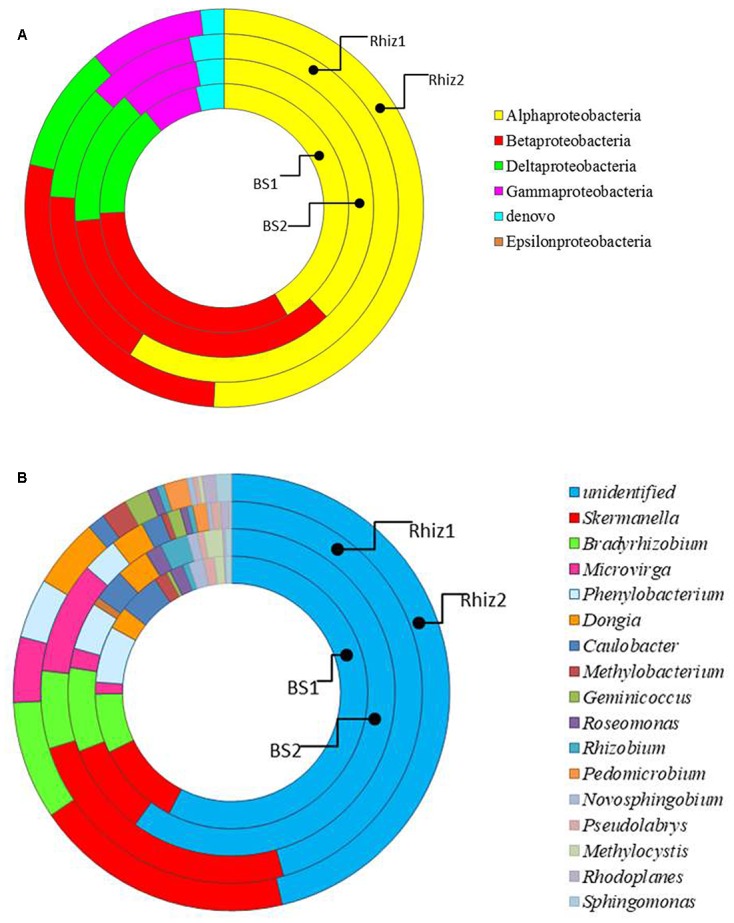
Distribution of the **(A)** phylum Proteobacteria and **(B)** genera belonging to alpha Proteobacteria in the different classes in bulk soil and rhizosphere of *V. vinifera* cv. Pinot Noir during the two sampling dates (flowering and early fruiting). From the center to the edge BS1, BS2, Rhiz1, Rhiz2.

Actinobacteria were the dominant class of bacteria with frequencies higher than 50% in all the soil samples, followed by alpha Proteobacteria, whose frequency in the rhizosphere significantly varied with time (Rhiz1 21.75%; Rhiz2 17.73%; *p* = 0.032) (**Figure 4**). *Gaiella*, *Arthrobacter*, and *Solirubrobacter* were the dominant identified genera belonging to Actinobacteria (**Figure 5**). The distribution of the different classes of Proteobacteria is reported in **Figure 6A**. Among Alpha Proteobacteria, *Skermanella* was the dominant identified genus (BS1 9.9%; BS2 8.8%; Rhiz1 24.0%; Rhiz2 18.9%) followed by *Bradyrhizobium* (**Figure 6B**). Beta Proteobacteria were similarly distributed in all the samples (BS1 8.02%; BS2 9.73%; Rhiz1 6.25%; and Rhiz2 9.65%). On the other hand, the abundance of Gamma Proteobacteria at the first sampling differed between bulk soil and rhizosphere (BS1 1.75%; Rhiz1 2.34%; *p* = 0.032) (**Figure 4**).

The most dominant genera were unclassified, belonging mainly to Actinobacteria, and their frequency did not change in the different the samples. *Gaiella* was the most represented identified genus in the class Actinobacteria, with a frequency varying between 2 and 5%. Dominant bacterial species were unclassified Actinomycetales, unclassified Solirubrobacterales and unclassified Micromonosporaceae whose frequency did not change in the various samples. On the contrary, the occurrence of unclassified Acidimicrobiales, unclassified Nocardioidaceae and unclassified Bradyrhizobiaceae differed between the bulk soil and the rhizosphere (*p* = 0.032, 0.016, 0.008), especially during the flowering. The list of bacterial species is reported in Supplementary Table 1.

**FIGURE 7:**
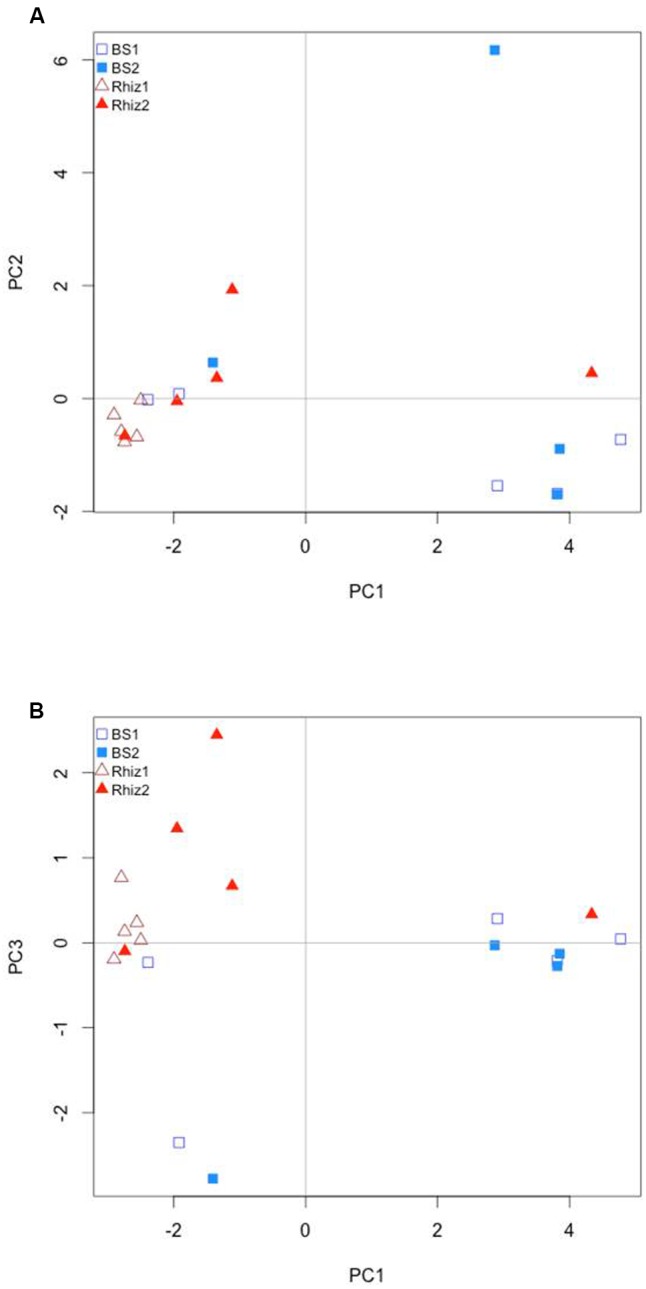
Comparison by principal component analysis of the ecological distance (Bray–Curtis) of the different compartments (bulk soil, rhizosphere soil) and the different harvest time (flowering and early fruit development) of *V. vinifera* cv. Pinot Noir: **(A)** axis 1 vs. axis 2; **(B)** axis 2 vs. axis 3.

PCA analysis revealed a different structure for the soil samples; in general, rhizosphere and bulk soil, regardless of the sampling date, were separated on the first axis. The bacterial community associated with the rhizosphere harvested at the early flowering time (Rhiz1) were clearly separated from all the other samples. This clustering was represented by axis1 (48.1%) that accounts for the highest amount of variability among the soil samples. Axis 2 accounts for about the 16% of variability, while axis 3 accounts for the 6% of variability (**Figures 7A,B**).

## Discussion

The possible effects of plant phenological stages and rhizodeposition on the composition of soil microbial communities have been widely studied especially in annual crops, and grasslands (Mougel et al., 2006; Houlden et al., 2008; Micallef et al., 2009; Xu et al., 2009; Chaparro et al., 2013a; Sugiyama et al., 2014). However, comparatively less information is available on soil microbial community composition in woody perennial agroecosystems such as vineyards (Steenwerth et al., 2008; Ulrich et al., 2008; Bulgari et al., 2009; Bokulich et al., 2014; Pinto et al., 2014). Several papers dealing with different crops reported that together with plant taxon (up to the species or even cultivar level) and soil type, agricultural practices are among the most important factors affecting the composition of the rhizosphere microbiome (Wu et al., 2008; Aranda et al., 2011; İnceoğlu et al., 2012; Dias et al., 2013; Jiang et al., 2017; Wen et al., 2017). To our knowledge, there has been only one previous report, obtained by phospholipid fatty acid analysis (PFLA) on the diversity of the rhizospheric microbial community of grapevine belonging to the cultivar Pinot Noir (Steenwerth et al., 2008). Microbial communities in vineyards, especially those subjected to IPM can be affected due to the presence of fertilizers, pesticides herbicides, however, only one manuscript reported the community structure of bacterial endophytes in a vineyard subjected to different pest management regimens (organic and IPM) (Campisano et al., 2014a).

In this work we: (i) characterized the microbiome of grapevine rhizosphere at two time points corresponding to flowering and early fruit development and (ii) this information was compared with the microbial community structure of the bulk soil. The use of a metagenomic approach allowed us to fully explore the rhizospheric diversity of bacterial communities of *V. vinifera* cv. Pinot Noir in a vineyard subjected to IPM.

There is a general consensus that root exudates can affect the structure of rhizosphere microbial communities (Bais et al., 2006; Haichar et al., 2008; Huang et al., 2014). Moreover, in annual plants, seedlings produce low levels of root exudates; the amount of root exudates gradually increases until the flowering stage and decreases again when the plant reaches maturity (Aulakh et al., 2001). During seedling development roots release mainly sugars that become substrates for a wide diversity of microbes. When the plant ages, other molecules, that may be able to select specific microbial inhabitants of the rhizosphere are released by the roots (Badri et al., 2013; Chaparro et al., 2013b).

On the contrary, our results showed variations of the microbial biodiversity influenced by the presence of the plant, but not by its phenological stage. The number of species observed in the rhizosphere was higher than that in the bulk soil at both sampling times. Consistently, microbial biodiversity measured as Shannon’s Index was higher in the rhizosphere than in the bulk soil. Moreover, PCA showed that bacterial communities changed significantly according to the presence of the plant (bulk soil vs. rhizosphere). This is consistent with several studies on other plant species describing different bacterial community structure for bulk and rhizosphere soil (Smalla et al., 2001; de Ridder-Duine et al., 2005; Breidenbach et al., 2016; Yang et al., 2017). Taken together, these results suggest that the rhizosphere effect is dominant over the phenological stage in determining overall microbial community patterns in the rhizosphere. Moreover, the impact of the rhizosphere effect appears to be more pronounced during flowering than during early fruit development.

Regarding the phyla distribution our results showed that, in all of the samples, the dominant phyla were: Actinobacteria (with an unusually high frequency, i.e., ≥50%), Proteobacteria, Gemmatimonadetes, and Bacteroidetes. This is in partial agreement with data recently reported by other studies (Opsi et al., 2014; Zarraonaindia et al., 2015). According to Opsi et al. (2014), Proteobacteria (36%), followed by Actinobacteria (26%) and Acidobacteria (15%) have been described to be the prevalent phyla in a vineyard located in Aosta Valley (north–west of Italy). Similarly, Zarraonaindia et al. (2015) analyzing bulk soil and grapevine (*V. vinifera* cv. Merlot) root samples harvested in five vineyards subjected to the same management in Long Island observed a dominance of sequences ascribed to Proteobacteria (32 and 57%, respectively), *Acidobacteria* spp. (19% in soil; 10% in root), *Bacteroidetes* spp. (10% in soil; 13% in root), and *Verrucomicrobia* spp. (8% in soil; 5% in root), with a greater relative abundance of *Planctomycetes* spp. in soils (7%) and of *Actinobacteria* spp. in roots (5.1%).

Actinobacteria and Proteobacteria are well-known as dominant phyla in soil. They are actively involved in carbon cycling and production of secondary metabolites (Jenkins et al., 2009). Actinobacteria are considered as oligotrophic K-strategists (Fierer et al., 2003), having slow growth, low nutritional requirements and high affinity for complex molecules. Therefore, the abundance of k-strategists overcomes that of r-strategists (characterized by fast growth and by a high affinity for simple carbon molecules), especially when the availability of organic carbon is low and the carbon inputs, also those deriving from fertilizers and pesticides, is reduced (Panikov, 1999). This is consistent with the low amount of total organic carbon (0.43%) measured in vineyard soil considered in the present study; moreover, it should be considered that the IPM provides a lower amount of chemical inputs such as pesticides and fertilizers that can, stimulate the growth of copiotrophic microorganisms. Proteobacteria include organisms with a wide variety of metabolic capabilities; members of alpha, beta, gamma, and delta -Proteobacteria, are commonly reported in soil. Members of the Alpha, Beta, and Gamma classes are considered to be copiotrophs (r-strategist), and they are prevalent where resource availability is high, such as in rhizosphere soils (Fierer et al., 2007). In the soils examined in this study, this was true for gamma Proteobacteria, but not for the other classes of bacteria.

Interestingly, the occurrence of members belonging to the phylum Gemmatimonadetes in vineyard soils has not been reported previously. However, the presence of sequences of Gemmatimonadetes are often observed in environmental 16S rRNA gene libraries; it has been estimated that this phylum represents one of the top nine phyla commonly found in soils, representing about 2% of soil bacterial diversity (Janssen, 2006). More recently, this information has been confirmed by DeBruyn et al. (2011) using high-throughput sequencing: according to these estimates Gemmatimonadetes relative abundances in large libraries (>500 sequences) from soils range from 0.2 to 6.5%, with a mean of 2.2%. Our results showed that the abundance of sequences ascribed to Gemmatimonadetes (6937 in total) ranged from 4% in rhizosphere to 8% in bulk soil. While most of the Gemmatimonadetes have been identified only at the genus level, about 32 sequences were ascribed to *Gemmatimonas aurantiaca* (24 coming from bulk soil) and 8 were ascribed to the strain *G. aurantiaca* T27 (7 of which were coming from the rhizosphere soil). This species has been described by Zhang et al. (2003) as a polyphosphate-accumulating strain isolated from wastewater; the greatest number of Gemmatimonadetes were detected in arid soils with a neutral pH (DeBruyn et al., 2011).

*Gaiella* was the dominant genus of Actinobacteria in all the samples. It forms non-motile rod-shaped cells that stain Gram-negative. These microorganisms are strictly aerobic, oxidase and catalase positive, and the type species is *Gaiella occulta*, described for the first time by Albuquerque et al. (2011). Consequently, very little information is available for members of this genus and studies of other genera phylogenetically close to *Gaiella* may be useful to improve our knowledge of the behavior and the response of this genus.

Our results showed the presence of lactic acid bacteria belonging to the family Lactobacillaceae such as *Lactobacillus iners* (data not shown). However, only three sequences corresponding to this species were detected in the rhizosphere of *V. vinifera* cv. Pinot Noir, thus suggesting that soil does not represent a favorable ecological niche or reservoir for microorganisms that may be involved in wine production (Chen et al., 2005; Zarraonaindia et al., 2015; Castañeda and Barbosa, 2016).

The occurrence of human opportunistic pathogens in the rhizosphere and in bulk soils has been described many times (Berg et al., 2005, 2014; Mendes et al., 2013; Campisano et al., 2014b) and attention has been paid especially to the pathogens able to colonize the plant internal tissues (Tyler and Triplett, 2008). Surprisingly, we did not detect any sequences corresponding to possible human or plant pathogens. Moreover, we found a median value of only six sequences ascribed to typical plant growth-promoting bacteria such as pseudomonads (data not shown). However, in a further study (Bona et al., under revision), we have analyzed the metaproteome of the soil bacterial community of the same vineyard at the flowering time. The results showed that the 15 (5.64%) and 19 (8.55%) of the proteins expressed in the bulk and rhizosphere soil, respectively, were synthesized by members of the genus Pseudomonas. Therefore, although not very represented, the pseudomonads population was rather active (Bona et al., under revision).

## Conclusion

Microbial community structures differed between bulk and rhizosphere soil, and this variability is not related to the plant phenological stage; this indicates that in the soils examined and with the cultivar *V. vinifera* cv. Pinot Noir, the effect of the factor “space” overcomes the effect of the factor “time.” However, a fraction of 94% (first sampling) and 97% (second sampling) of the sequences were shared between soil and rhizosphere samples suggesting that the soil is a reservoir of rhizospheric microorganisms.

The data presented in this work highlight the importance of studying the microbiome associated with grapevine and the need for a more detailed characterization of the plant microbe interactions. These results will contribute to the characterization of the biodiversity of grapevines and to the identification of possible biomarkers of typical features for *V. vinifera* cv. Pinot Noir.

## Author Contributions

GN, NM, PC, EB, and EG have been involved in soil sampling, DNA extraction and amplification; LB and FM performed pyrosequencing by Roche 454 and analyzed the data; GN and EG wrote the main manuscript text; GB and GL coordinated biological experiments, data analysis and paper writing. All authors revised the manuscript.

## Conflict of Interest Statement

The authors declare that the research was conducted in the absence of any commercial or financial relationships that could be construed as a potential conflict of interest.

## Footnotes

1 https://www.istat.it/it/archivio/194422

2 http://whc.unesco.org/en/list/1390

3 https://rdp.cme.msu.edu

4 https://www.r-project.org/
