# Supplementary material for: The Rhizosphere Bacterial Microbiota of Vitis vinifera cv. Pinot Noir in an Integrated Pest Management Vineyard
Source: Front Microbiol. 2017 Aug 14;8:1528. doi: 10.3389/fmicb.2017.01528 (PMC5557794; doi:10.3389/fmicb.2017.01528)
Supplement: Supplementary file 1 [file Table_1.DOC]

**Title:** The rhizosphere bacterial microbiota of *Vitis vinifera* cv. Pinot noir in an integrated pest management vineyard.

**Author list:** Novello G.^1^, Gamalero E. ^1*^, Bona E. ^1^, Boatti L.^1,2^, Mignone F.^1,2^, Massa N. ^1^, Cesaro P ^1^, Lingua G. ^1^, Berta G. ^1^

**Table 1.** Full list of bacterial species and their frequency in the different soil samples.

|  |  |  | |  |  |  |  | **FREQUENCY (%)** | | | | |  | |  | **P-value** | | | | | | | | |
| --- | --- | --- | --- | --- | --- | --- | --- | --- | --- | --- | --- | --- | --- | --- | --- | --- | --- | --- | --- | --- | --- | --- | --- | --- |
| **phylum** | **class** | **order** | | **family** | **genus** | **species** | BS1 | | BS2 | | Rhiz 1 | | Rhiz 2 | | | | BS1 vs Bs2 | | BS1 vs Rhiz1 | | BS2 vs Rhiz2 | | Rhiz1 vs Rhiz2 | |
| Actinobacteria | Actinobacteria | Actinomycetales | | unclassified | unclassified | unclassified | 7,75 | | 8,87 | | 4,61 | | 7,73 | | | | 0,905 | | 0,095 | | 0,325 | | 0,690 | |
| Actinobacteria | Actinobacteria | Solirubrobacterales | | unclassified | unclassified | unclassified | 5,36 | | 5,42 | | 6,07 | | 4,22 | | | | 0,712 | | 1,000 | | 0,286 | | 0,056 | |
| Actinobacteria | Actinobacteria | Actinomycetales | | Micromonosporaceae | unclassified | unclassified | 5,36 | | 7,20 | | 3,56 | | 5,17 | | | | 0,413 | | 0,249 | | 0,063 | | 0,841 | |
| Actinobacteria | Actinobacteria | unclassified | | unclassified | unclassified | unclassified | 3,43 | | 2,65 | | 3,86 | | 4,28 | | | | 0,556 | | 0,151 | | 0,556 | | 0,222 | |
| Proteobacteria | Betaproteobacteria | unclassified | | unclassified | unclassified | unclassified | 2,90 | | 4,46 | | 2,48 | | 2,97 | | | | 0,556 | | 1,000 | | 0,413 | | 0,548 | |
| Actinobacteria | Actinobacteria | Gaiellales | | Gaiellaceae | Gaiella | bacterium Ellin6526 | 5,78 | | 2,82 | | 1,47 | | 1,66 | | | | 0,413 | | 0,116 | | 0,730 | | 1,000 | |
| Proteobacteria | Alphaproteobacteria | Rhizobiales | | unclassified | unclassified | unclassified | 2,08 | | 1,99 | | 4,18 | | 3,12 | | | | 0,806 | | 0,222 | | 0,325 | | 0,548 | |
| Actinobacteria | unclassified | unclassified | | unclassified | unclassified | unclassified | 3,03 | | 3,17 | | 2,49 | | 2,40 | | | | 0,730 | | 0,675 | | 0,190 | | 0,094 | |
| Proteobacteria | Deltaproteobacteria | Myxococcales | | unclassified | unclassified | unclassified | 1,68 | | 2,23 | | 2,14 | | 2,65 | | | | 1,000 | | 0,209 | | 0,730 | | 0,421 | |
| Actinobacteria | Actinobacteria | Acidimicrobiales | | unclassified | unclassified | unclassified | 1,07 | | 1,80 | | 2,48 | | 2,64 | | | | 0,556 | | 0,032 | | 1,000 | | 0,463 | |
| Gemmatimonadetes | Gemmatimonadetes | Gemmatimonadales | | Gemmatimonadaceae | Gemmatimonas | Gemmatimonadaceae bacterium LWQ133 | 2,14 | | 3,37 | | 1,07 | | 0,97 | | | | 0,730 | | 0,056 | | 0,730 | | 0,548 | |
| Actinobacteria | Actinobacteria | Actinomycetales | | Nocardioidaceae | unclassified | unclassified | 1,73 | | 1,97 | | 1,92 | | 1,63 | | | | 1,000 | | 0,016 | | 0,730 | | 0,600 | |
| Proteobacteria | Alphaproteobacteria | unclassified | | unclassified | unclassified | unclassified | 0,94 | | 1,30 | | 1,86 | | 2,16 | | | | 0,556 | | 0,016 | | 0,730 | | 0,310 | |
| Proteobacteria | Betaproteobacteria | Burkholderiales | | Burkholderiales_incertae_sedis | unclassified | unclassified | 1,48 | | 1,40 | | 1,56 | | 1,79 | | | | 0,905 | | 0,222 | | 1,000 | | 0,548 | |
| Gemmatimonadetes | Gemmatimonadetes | Gemmatimonadales | | Gemmatimonadaceae | Gemmatimonas | unidentified bacterium | 1,43 | | 1,39 | | 1,12 | | 1,66 | | | | 0,730 | | 0,310 | | 0,730 | | 0,917 | |
| Actinobacteria | Actinobacteria | Actinomycetales | | Intrasporangiaceae | unclassified | unclassified | 1,22 | | 1,39 | | 1,11 | | 0,88 | | | | 0,730 | | 0,462 | | 0,325 | | 0,059 | |
| Actinobacteria | Actinobacteria | Gaiellales | | Gaiellaceae | Gaiella | Gaiella occulta (T) | 0,61 | | 0,32 | | 1,73 | | 1,79 | | | | 0,461 | | 0,421 | | 0,413 | | 0,548 | |
| Proteobacteria | unclassified | unclassified | | unclassified | unclassified | unclassified | 1,10 | | 1,07 | | 1,29 | | 0,95 | | | | 1,000 | | 0,036 | | 0,712 | | 0,548 | |
| Proteobacteria | Alphaproteobacteria | Rhizobiales | | Bradyrhizobiaceae | unclassified | unclassified | 0,70 | | 0,71 | | 1,50 | | 1,29 | | | | 0,730 | | 0,008 | | 0,712 | | 0,209 | |
| Gemmatimonadetes | Gemmatimonadetes | Gemmatimonadales | | Gemmatimonadaceae | Gemmatimonas | Gemmatimonadetes bacterium KBS708 | 2,05 | | 1,65 | | 0,18 | | 0,28 | | | | 1,000 | | 0,142 | | 0,389 | | 0,834 | |
| Actinobacteria | Actinobacteria | Actinomycetales | | Geodermatophilaceae | unclassified | unclassified | 1,31 | | 1,63 | | 0,46 | | 0,77 | | | | 1,000 | | 0,346 | | 0,413 | | 0,346 | |
| Proteobacteria | Deltaproteobacteria | Myxococcales | | Polyangiaceae | unclassified | unclassified | 0,65 | | 0,95 | | 0,98 | | 1,55 | | | | 0,730 | | 0,421 | | 0,730 | | 1,000 | |
| Proteobacteria | Alphaproteobacteria | Rhodospirillales | | Acetobacteraceae | unclassified | unclassified | 1,49 | | 1,29 | | 0,48 | | 0,62 | | | | 1,000 | | 0,401 | | 0,176 | | 0,833 | |
| Proteobacteria | Betaproteobacteria | Burkholderiales | | unclassified | unclassified | unclassified | 0,90 | | 0,64 | | 0,88 | | 1,33 | | | | 0,905 | | 0,690 | | 0,286 | | 0,222 | |
| Actinobacteria | Actinobacteria | Actinomycetales | | Geodermatophilaceae | Blastococcus | bacterium Ellin6023 | 1,31 | | 1,10 | | 0,49 | | 0,76 | | | | 0,730 | | 0,008 | | 0,905 | | 0,548 | |
| Proteobacteria | Gammaproteobacteria | unclassified | | unclassified | unclassified | unclassified | 0,80 | | 0,60 | | 0,80 | | 1,28 | | | | 0,712 | | 0,295 | | 0,556 | | 1,000 | |
| Actinobacteria | Actinobacteria | Gaiellales | | Gaiellaceae | Gaiella | agricultural soil bacterium SC-I-92 | 2,00 | | 0,92 | | 0,18 | | 0,29 | | | | 0,413 | | 0,548 | | 0,806 | | 0,917 | |
| Bacteroidetes | Sphingobacteriia | Sphingobacteriales | | Chitinophagaceae | unclassified | unclassified | 0,69 | | 0,81 | | 0,93 | | 0,92 | | | | 0,539 | | 0,094 | | 1,000 | | 1,000 | |
| Actinobacteria | Actinobacteria | Solirubrobacterales | | Solirubrobacteraceae | Solirubrobacter | Solirubrobacter ginsenosidimutans | 0,82 | | 0,26 | | 1,29 | | 0,67 | | | | 0,323 | | 0,116 | | 0,323 | | 0,047 | |
| Proteobacteria | Betaproteobacteria | Burkholderiales | | Comamonadaceae | unclassified | unclassified | 1,01 | | 0,89 | | 0,56 | | 0,68 | | | | 0,905 | | 0,151 | | 0,016 | | 0,310 | |
| Proteobacteria | Betaproteobacteria | Burkholderiales | | Oxalobacteraceae | unclassified | unclassified | 0,91 | | 0,69 | | 0,32 | | 1,26 | | | | 0,730 | | 0,295 | | 0,905 | | 0,295 | |
| Actinobacteria | Actinobacteria | Gaiellales | | Gaiellaceae | Gaiella | bacterium Ellin7530 | 1,73 | | 0,92 | | 0,12 | | 0,24 | | | | 0,413 | | 0,095 | | 0,623 | | 0,346 | |
| Actinobacteria | Actinobacteria | Actinomycetales | | Micrococcaceae | unclassified | unclassified | 0,87 | | 1,09 | | 0,29 | | 0,56 | | | | 1,000 | | 0,020 | | 0,556 | | 0,590 | |
| Proteobacteria | Alphaproteobacteria | Rhodospirillales | | Rhodospirillaceae | Skermanella | Skermanella aerolata | 0,24 | | 0,38 | | 1,16 | | 0,73 | | | | 1,000 | | 0,151 | | 0,806 | | 0,222 | |
| Proteobacteria | Alphaproteobacteria | Rhodospirillales | | Rhodospirillaceae | Skermanella | Skermanella sp. JC5 | 0,38 | | 0,19 | | 1,10 | | 0,79 | | | | 0,537 | | 0,095 | | 0,711 | | 0,249 | |
| Actinobacteria | Actinobacteria | Actinomycetales | | Geodermatophilaceae | Blastococcus | Blastococcus sp. OS1-29 | 1,06 | | 0,99 | | 0,16 | | 0,21 | | | | 0,905 | | 0,295 | | 0,268 | | 0,671 | |
| Actinobacteria | Actinobacteria | Actinomycetales | | Pseudonocardiaceae | unclassified | unclassified | 0,51 | | 0,70 | | 0,66 | | 0,51 | | | | 0,730 | | 0,548 | | 0,905 | | 0,463 | |
| Actinobacteria | Actinobacteria | Solirubrobacterales | | Solirubrobacteraceae | Solirubrobacter | bacterium Ellin6048 | 0,16 | | 0,07 | | 1,09 | | 0,96 | | | | 0,387 | | 0,032 | | 0,140 | | 0,222 | |
| Proteobacteria | Gammaproteobacteria | Xanthomonadales | | Xanthomonadaceae | unclassified | unclassified | 0,23 | | 0,75 | | 0,63 | | 0,74 | | | | 0,111 | | 0,047 | | 0,556 | | 0,690 | |
| Actinobacteria | Actinobacteria | Gaiellales | | Gaiellaceae | Gaiella | bacterium Ellin7545 | 1,02 | | 0,93 | | 0,14 | | 0,22 | | | | 0,730 | | 0,075 | | 0,556 | | 0,421 | |
| Proteobacteria | Alphaproteobacteria | Rhodospirillales | | unclassified | unclassified | unclassified | 0,30 | | 0,41 | | 0,71 | | 0,81 | | | | 0,902 | | 0,059 | | 0,032 | | 0,841 | |
| Proteobacteria | Alphaproteobacteria | Rhodospirillales | | Rhodospirillaceae | Skermanella | Azospirillum sp. AP-500 | 0,19 | | 0,23 | | 0,95 | | 0,70 | | | | 1,000 | | 0,249 | | 0,806 | | 0,249 | |
| Proteobacteria | Alphaproteobacteria | Rhizobiales | | Bradyrhizobiaceae | Bradyrhizobium | Bradyrhizobium sp. WSM471 | 0,30 | | 0,47 | | 0,56 | | 0,72 | | | | 0,712 | | 0,056 | | 0,621 | | 0,463 | |
| Actinobacteria | Actinobacteria | Solirubrobacterales | | Solirubrobacteraceae | Solirubrobacter | Solirubrobacter sp. Gsoil 917 | 0,33 | | 0,27 | | 0,78 | | 0,54 | | | | 0,730 | | 0,056 | | 0,459 | | 0,142 | |
| Gemmatimonadetes | Gemmatimonadetes | Gemmatimonadales | | Gemmatimonadaceae | Gemmatimonas | Gemmatimonadetes bacterium Ellin7146 | 0,62 | | 0,60 | | 0,35 | | 0,37 | | | | 1,000 | | 0,675 | | 0,905 | | 0,462 | |
| Proteobacteria | Gammaproteobacteria | Xanthomonadales | | Sinobacteraceae | Steroidobacter | agricultural soil bacterium SC-I-87 | 0,13 | | 0,14 | | 0,90 | | 0,59 | | | | 0,806 | | 0,016 | | 0,190 | | 0,310 | |
| Actinobacteria | Actinobacteria | Actinomycetales | | Microbacteriaceae | unclassified | unclassified | 0,36 | | 0,50 | | 0,53 | | 0,35 | | | | 0,902 | | 0,675 | | 0,905 | | 0,222 | |
| Acidobacteria | Acidobacteria_Gp1 | unclassified | | unclassified | unclassified | unclassified | 0,75 | | 0,51 | | 0,00 | | 0,22 | | | | 1,000 | | 0,106 | | 0,621 | | 0,265 | |
| Actinobacteria | Actinobacteria | Acidimicrobiales | | Iamiaceae | Iamia | bacterium Ellin5273 | 0,19 | | 0,09 | | 0,61 | | 0,43 | | | | 0,413 | | 0,047 | | 0,413 | | 0,047 | |
| Actinobacteria | Actinobacteria | Acidimicrobiales | | Acidimicrobiaceae | Ilumatobacter | unidentified marine bacterioplankton | 0,02 | | 0,00 | | 0,73 | | 0,56 | | | | 0,199 | | 0,016 | | 0,125 | | 0,008 | |
| Gemmatimonadetes | Gemmatimonadetes | Gemmatimonadales | | Gemmatimonadaceae | Gemmatimonas | Gemmatimonadetes bacterium LX87 | 0,33 | | 0,13 | | 0,49 | | 0,35 | | | | 0,190 | | 0,222 | | 0,268 | | 0,142 | |
| Actinobacteria | Actinobacteria | Actinomycetales | | Intrasporangiaceae | Intrasporangium | Intrasporangium calvum DSM 43043 | 0,37 | | 0,43 | | 0,31 | | 0,11 | | | | 0,905 | | 0,753 | | 0,266 | | 0,059 | |
| Actinobacteria | Actinobacteria | Actinomycetales | | Intrasporangiaceae | Tetrasphaera | Tetrasphaera sp. Ellin115 | 0,50 | | 0,22 | | 0,27 | | 0,18 | | | | 0,325 | | 0,917 | | 0,902 | | 0,173 | |
| Gemmatimonadetes | Gemmatimonadetes | Gemmatimonadales | | Gemmatimonadaceae | Gemmatimonas | Microcystis sp. SAG 43.90 | 0,37 | | 0,69 | | 0,03 | | 0,13 | | | | 1,000 | | 0,008 | | 0,389 | | 0,074 | |
| Acidobacteria | Acidobacteria_Gp3 | Gp3 | | Gp3 | Gp3 | unidentified bacterium | 0,47 | | 0,23 | | 0,14 | | 0,35 | | | | 0,325 | | 0,310 | | 1,000 | | 0,600 | |
| Actinobacteria | Actinobacteria | Gaiellales | | Gaiellaceae | Gaiella | bacterium Ellin7507 | 0,55 | | 0,36 | | 0,11 | | 0,09 | | | | 0,389 | | 0,075 | | 0,900 | | 0,916 | |
| Bacteroidetes | Flavobacteriia | Flavobacteriales | | Flavobacteriaceae | Chryseobacterium | Chryseobacterium sp. CI02 | 0,24 | | 0,69 | | 0,12 | | 0,10 | | | | 0,901 | | 0,600 | | 0,901 | | 0,834 | |
| Actinobacteria | Actinobacteria | Solirubrobacterales | | Solirubrobacteraceae | Solirubrobacter | bacterium Ellin5249 | 0,08 | | 0,09 | | 0,50 | | 0,39 | | | | 0,533 | | 0,095 | | 0,211 | | 0,222 | |
| Actinobacteria | Actinobacteria | Actinomycetales | | Kineosporiaceae | Kineosporia | Kineosporia rhamnosa (T) | 0,28 | | 0,39 | | 0,21 | | 0,20 | | | | 0,539 | | 0,675 | | 0,556 | | 0,674 | |
| Actinobacteria | Actinobacteria | Actinomycetales | | Streptomycetaceae | unclassified | unclassified | 0,27 | | 0,22 | | 0,11 | | 0,50 | | | | 0,730 | | 0,295 | | 0,413 | | 0,205 | |
| Actinobacteria | Actinobacteria | Actinomycetales | | Nocardioidaceae | Marmoricola | Marmoricola sp. Gsoil 818 | 0,30 | | 0,33 | | 0,24 | | 0,15 | | | | 1,000 | | 0,402 | | 0,623 | | 0,675 | |
| Actinobacteria | Actinobacteria | Solirubrobacterales | | Conexibacteraceae | Conexibacter | bacterium Ellin504 | 0,28 | | 0,39 | | 0,17 | | 0,20 | | | | 0,268 | | 0,248 | | 0,286 | | 0,528 | |
| Actinobacteria | Actinobacteria | Actinomycetales | | Micrococcaceae | Arthrobacter | Arthrobacter sp. R40S | 0,03 | | 0,01 | | 0,27 | | 0,77 | | | | 0,453 | | 0,116 | | 0,133 | | 0,548 | |
| Acidobacteria | Acidobacteria_Gp3 | Gp3 | | Gp3 | Gp3 | bacterium enrichment culture clone SC-2_22 | 0,45 | | 0,21 | | 0,14 | | 0,20 | | | | 0,556 | | 0,222 | | 1,000 | | 1,000 | |
| Proteobacteria | Alphaproteobacteria | Rhodospirillales | | Rhodospirillaceae | Dongia | Rhodospirillales bacterium WX36 | 0,03 | | 0,00 | | 0,43 | | 0,53 | | | | 0,502 | | 0,142 | | 0,073 | | 1,000 | |
| Acidobacteria | Acidobacteria_Gp10 | Gp10 | | Gp10 | Gp10 | Acidobacteria bacterium WY11 | 0,10 | | 0,05 | | 0,37 | | 0,45 | | | | 0,621 | | 0,012 | | 0,190 | | 0,421 | |
| Proteobacteria | Alphaproteobacteria | Rhodospirillales | | Rhodospirillaceae | unclassified | unclassified | 0,14 | | 0,12 | | 0,39 | | 0,29 | | | | 0,905 | | 0,095 | | 0,905 | | 0,016 | |
| Actinobacteria | Actinobacteria | Solirubrobacterales | | Conexibacteraceae | Conexibacter | Rubrobacterineae bacterium BR7-21 | 0,46 | | 0,33 | | 0,07 | | 0,11 | | | | 0,905 | | 0,010 | | 0,539 | | 0,239 | |
| Proteobacteria | Alphaproteobacteria | Rhodospirillales | | Rhodospirillaceae | Skermanella | Azospirillum sp. enrichment culture clone VanCtr101 | 0,13 | | 0,05 | | 0,47 | | 0,26 | | | | 0,806 | | 0,548 | | 0,623 | | 0,151 | |
| Gemmatimonadetes | Gemmatimonadetes | Gemmatimonadales | | Gemmatimonadaceae | Gemmatimonas | bacterium Ellin5290 | 0,35 | | 0,26 | | 0,14 | | 0,21 | | | | 0,806 | | 0,527 | | 1,000 | | 0,916 | |
| Actinobacteria | Actinobacteria | Gaiellales | | Gaiellaceae | Gaiella | actinobacterium WWH12 | 0,05 | | 0,02 | | 0,52 | | 0,32 | | | | 0,137 | | 0,209 | | 0,262 | | 0,421 | |
| Proteobacteria | Deltaproteobacteria | unclassified | | unclassified | unclassified | unclassified | 0,24 | | 0,07 | | 0,27 | | 0,35 | | | | 0,806 | | 0,463 | | 0,461 | | 0,841 | |
| Proteobacteria | Alphaproteobacteria | Rhodospirillales | | Rhodospirillaceae | Skermanella | Azospirillum sp. LH-CAB12 | 0,05 | | 0,01 | | 0,63 | | 0,18 | | | | 0,898 | | 0,142 | | 0,533 | | 0,056 | |
| Actinobacteria | Actinobacteria | Actinomycetales | | Micrococcaceae | Arthrobacter | Arthrobacter globiformis | 0,15 | | 0,14 | | 0,25 | | 0,41 | | | | 0,902 | | 0,458 | | 0,461 | | 0,173 | |
| Gemmatimonadetes | Gemmatimonadetes | Gemmatimonadales | | Gemmatimonadaceae | Gemmatimonas | bacterium enrichment culture clone auto9_4W | 0,40 | | 0,35 | | 0,07 | | 0,12 | | | | 0,806 | | 0,046 | | 0,730 | | 0,462 | |
| Actinobacteria | Actinobacteria | Actinomycetales | | Micrococcaceae | Arthrobacter | Arthrobacter sp. S88 | 0,21 | | 0,73 | | 0,00 | | 0,00 | | | | 0,533 | | 0,158 | | 0,201 | | 0,519 | |
| Proteobacteria | Deltaproteobacteria | Myxococcales | | Cystobacteraceae | unclassified | unclassified | 0,16 | | 0,42 | | 0,16 | | 0,15 | | | | 0,082 | | 0,599 | | 0,268 | | 0,600 | |
| Proteobacteria | Alphaproteobacteria | Rhizobiales | | Methylobacteriaceae | Microvirga | Microvirga zambiensis (T) | 0,00 | | 0,00 | | 0,55 | | 0,26 | | | | 0,561 | | 0,034 | | 0,073 | | 0,032 | |
| Actinobacteria | Actinobacteria | Actinomycetales | | Nakamurellaceae | Nakamurella | Humicoccus sp. L1886 | 0,05 | | 0,05 | | 0,50 | | 0,15 | | | | 0,806 | | 0,095 | | 0,266 | | 0,116 | |
| Actinobacteria | Actinobacteria | Actinomycetales | | Geodermatophilaceae | Blastococcus | Blastococcus sp. L1961 | 0,28 | | 0,22 | | 0,14 | | 0,15 | | | | 0,461 | | 0,249 | | 0,905 | | 0,598 | |
| Proteobacteria | Alphaproteobacteria | Rhizobiales | | Methylobacteriaceae | Microvirga | alpha proteobacterium TP1 | 0,00 | | 0,01 | | 0,44 | | 0,29 | | | | 0,893 | | 0,070 | | 0,171 | | 0,344 | |
| Actinobacteria | Actinobacteria | Gaiellales | | Gaiellaceae | Gaiella | bacterium enrichment culture clone F44x_D7_T3_E05 | 0,23 | | 0,11 | | 0,18 | | 0,23 | | | | 0,413 | | 1,000 | | 0,413 | | 0,753 | |
| Actinobacteria | Actinobacteria | Actinomycetales | | Pseudonocardiaceae | Pseudonocardia | Pseudonocardia sp. CNS139 PL04 | 0,19 | | 0,04 | | 0,23 | | 0,28 | | | | 0,902 | | 1,000 | | 0,706 | | 0,205 | |
| Acidobacteria | Acidobacteria_Gp3 | unclassified | | unclassified | unclassified | unclassified | 0,25 | | 0,21 | | 0,07 | | 0,21 | | | | 0,325 | | 0,310 | | 1,000 | | 0,600 | |
| Actinobacteria | Actinobacteria | Actinomycetales | | Geodermatophilaceae | Blastococcus | Blastococcus aggregatus | 0,21 | | 0,14 | | 0,14 | | 0,24 | | | | 0,323 | | 0,141 | | 0,806 | | 0,675 | |
| Proteobacteria | Alphaproteobacteria | Rhodospirillales | | Rhodospirillaceae | Skermanella | Skermanella sp. B-121-1-2 | 0,02 | | 0,05 | | 0,42 | | 0,18 | | | | 0,802 | | 0,021 | | 0,621 | | 0,036 | |
| Actinobacteria | Actinobacteria | Actinomycetales | | Nocardioidaceae | Nocardioides | Nocardioides sp. S23405 | 0,21 | | 0,21 | | 0,14 | | 0,12 | | | | 0,802 | | 0,021 | | 0,621 | | 0,036 | |
| Acidobacteria | Acidobacteria_Gp7 | Gp7 | | Gp7 | Gp7 | unidentified bacterium | 0,03 | | 0,02 | | 0,26 | | 0,38 | | | | 0,321 | | 0,142 | | 0,105 | | 1,000 | |
| Actinobacteria | Actinobacteria | Actinomycetales | | Pseudonocardiaceae | Pseudonocardia | Pseudonocardia sp. ACT-0146 | 0,10 | | 0,03 | | 0,25 | | 0,29 | | | | 0,902 | | 0,421 | | 0,623 | | 0,310 | |
| Bacteroidetes | Bacteroidetes_incertae_sedis | Ohtaekwangia | | Ohtaekwangia | Ohtaekwangia | Sphingobacteria bacterium RYG | 0,03 | | 0,07 | | 0,34 | | 0,21 | | | | 0,802 | | 0,046 | | 0,105 | | 0,462 | |
| Actinobacteria | Actinobacteria | Actinomycetales | | Microbacteriaceae | Agromyces | Agromyces ramosus (T) | 0,00 | | 0,01 | | 0,35 | | 0,28 | | | | 1,000 | | 0,044 | | 0,105 | | 0,346 | |
| Acidobacteria | Acidobacteria_Gp3 | Gp3 | | Gp3 | Gp3 | Acidobacteria bacterium LWQ4 | 0,21 | | 0,13 | | 0,16 | | 0,15 | | | | 0,387 | | 0,834 | | 1,000 | | 0,243 | |
| Proteobacteria | Alphaproteobacteria | Rhodospirillales | | Rhodospirillaceae | Skermanella | Skermanella sp. VTT E-073090 | 0,05 | | 0,03 | | 0,37 | | 0,13 | | | | 0,802 | | 0,222 | | 0,532 | | 0,139 | |
| Gemmatimonadetes | Gemmatimonadetes | Gemmatimonadales | | Gemmatimonadaceae | Gemmatimonas | Gemmatimonas aurantiaca | 0,19 | | 0,27 | | 0,02 | | 0,16 | | | | 0,902 | | 0,206 | | 1,000 | | 0,091 | |
| Proteobacteria | Deltaproteobacteria | Myxococcales | | Kofleriaceae | Kofleria | Kofleria flava | 0,17 | | 0,13 | | 0,13 | | 0,18 | | | | 0,623 | | 0,600 | | 1,000 | | 1,000 | |
| Actinobacteria | Actinobacteria | Gaiellales | | Gaiellaceae | Gaiella | unidentified bacterium | 0,32 | | 0,10 | | 0,06 | | 0,12 | | | | 0,140 | | 0,071 | | 1,000 | | 0,340 | |
| Proteobacteria | Alphaproteobacteria | Caulobacterales | | Caulobacteraceae | Phenylobacterium | Phenylobacterium sp. W2.09-62 | 0,11 | | 0,14 | | 0,17 | | 0,17 | | | | 0,902 | | 0,346 | | 0,711 | | 0,530 | |
| Actinobacteria | Actinobacteria | Actinomycetales | | Nocardioidaceae | Marmoricola | Nocardioides iriomotensis | 0,13 | | 0,19 | | 0,13 | | 0,15 | | | | 0,900 | | 0,599 | | 0,902 | | 0,548 | |
| Actinobacteria | Actinobacteria | Acidimicrobiales | | Acidimicrobiaceae | Ilumatobacter | unidentified bacterium | 0,07 | | 0,02 | | 0,32 | | 0,15 | | | | 0,217 | | 0,095 | | 0,133 | | 0,151 | |
| Proteobacteria | Alphaproteobacteria | Alphaproteobacteria_incertae_sedis | | Geminicoccus | Geminicoccus | alpha proteobacterium EXPOSESPACE_OU21 | 0,05 | | 0,01 | | 0,20 | | 0,34 | | | | 0,617 | | 0,075 | | 0,533 | | 1,000 | |
| Actinobacteria | Actinobacteria | Actinomycetales | | Micrococcaceae | Arthrobacter | Arthrobacter sp. M29 | 0,26 | | 0,30 | | 0,00 | | 0,02 | | | | 1,000 | | 0,025 | | 0,169 | | 0,071 | |
| Actinobacteria | Actinobacteria | Actinomycetales | | Streptomycetaceae | Streptomyces | Streptomyces canu | 0,07 | | 0,13 | | 0,17 | | 0,20 | | | | 1,000 | | 0,344 | | 0,900 | | 0,598 | |
| Actinobacteria | Actinobacteria | Actinomycetales | | Microbacteriaceae | Agromyces | Agromyces sp. L2361 | 0,02 | | 0,01 | | 0,39 | | 0,10 | | | | 0,797 | | 0,012 | | 0,163 | | 0,172 | |
| Proteobacteria | Betaproteobacteria | Burkholderiales | | Oxalobacteraceae | Noviherbaspirillum | Comamonadaceae bacterium MSCB-9 | 0,21 | | 0,28 | | 0,02 | | 0,06 | | | | 0,905 | | 0,070 | | 0,539 | | 0,136 | |
| Actinobacteria | Actinobacteria | Actinomycetales | | Nakamurellaceae | Nakamurella | Nakamurella flavida | 0,05 | | 0,16 | | 0,23 | | 0,10 | | | | 0,804 | | 0,093 | | 0,804 | | 0,293 | |
| Proteobacteria | Alphaproteobacteria | Sphingomonadales | | Erythrobacteraceae | unclassified | unclassified | 0,05 | | 0,24 | | 0,15 | | 0,12 | | | | 0,176 | | 0,141 | | 0,084 | | 0,059 | |
| Acidobacteria | Acidobacteria_Gp1 | Gp1 | | Gp1 | Gp1 | bacterium Ellin7522 | 0,35 | | 0,18 | | 0,00 | | 0,02 | | | | 0,260 | | 0,101 | | 1,000 | | 0,366 | |
| Proteobacteria | Alphaproteobacteria | Sphingomonadales | | Sphingomonadaceae | unclassified | unclassified | 0,16 | | 0,10 | | 0,13 | | 0,14 | | | | 0,712 | | 1,000 | | 0,537 | | 1,000 | |
| Actinobacteria | Actinobacteria | Solirubrobacterales | | Solirubrobacteraceae | Solirubrobacter | Solirubrobacter soli (T) | 0,10 | | 0,01 | | 0,24 | | 0,16 | | | | 0,104 | | 0,095 | | 0,019 | | 0,047 | |
| Actinobacteria | Actinobacteria | Actinomycetales | | Micromonosporaceae | Virgisporangium | Virgisporangium ochraceum | 0,02 | | 0,01 | | 0,34 | | 0,13 | | | | 0,797 | | 0,141 | | 0,453 | | 0,059 | |
| Actinobacteria | Actinobacteria | Acidimicrobiales | | Acidimicrobiaceae | Ilumatobacter | Ilumatobacter fluminis (T) | 0,02 | | 0,00 | | 0,30 | | 0,18 | | | | 0,348 | | 0,402 | | 0,443 | | 0,095 | |
| Actinobacteria | Actinobacteria | Actinomycetales | | Micromonosporaceae | Catelliglobosispora | Catelliglobosispora koreensis (T) | 0,05 | | 0,00 | | 0,30 | | 0,14 | | | | 0,500 | | 0,092 | | 0,348 | | 0,093 | |
| Actinobacteria | Actinobacteria | Gaiellales | | Gaiellaceae | Gaiella | Actinomycetales | 0,16 | | 0,09 | | 0,11 | | 0,15 | | | | 0,413 | | 1,000 | | 0,711 | | 0,673 | |
| Actinobacteria | Actinobacteria | Actinomycetales | | Streptomycetaceae | Streptomyces | Streptomyces viridochromogenes subsp. komabensis | 0,18 | | 0,28 | | 0,00 | | 0,04 | | | | 0,901 | | 0,045 | | 0,901 | | 0,057 | |
| Proteobacteria | Betaproteobacteria | Burkholderiales | | Burkholderiaceae | Burkholderia | Burkholderia sp. MS4t | 0,16 | | 0,27 | | 0,01 | | 0,04 | | | | 1,000 | | 0,387 | | 1,000 | | 0,588 | |
| Actinobacteria | Actinobacteria | Actinomycetales | | Nocardioidaceae | Marmoricola | Nocardioides sp. MTD22 | 0,05 | | 0,04 | | 0,20 | | 0,15 | | | | 0,711 | | 0,310 | | 0,387 | | 0,310 | |
| Proteobacteria | Alphaproteobacteria | Rhodospirillales | | Acetobacteraceae | Roseomonas | Acetobacteraceae bacterium GIMN 1.017 | 0,13 | | 0,14 | | 0,08 | | 0,10 | | | | 0,806 | | 1,000 | | 0,533 | | 0,674 | |
| Actinobacteria | Actinobacteria | Actinomycetales | | Nocardioidaceae | Nocardioides | Nocardioides islandensis (T) | 0,13 | | 0,10 | | 0,14 | | 0,06 | | | | 0,806 | | 0,600 | | 1,000 | | 0,310 | |
| Proteobacteria | Betaproteobacteria | Burkholderiales | | Oxalobacteraceae | Noviherbaspirillum | Oxalobacter sp. W1.09-142 | 0,22 | | 0,12 | | 0,04 | | 0,05 | | | | 0,533 | | 0,521 | | 0,623 | | 0,829 | |
| Proteobacteria | Betaproteobacteria | Burkholderiales | | Oxalobacteraceae | Noviherbaspirillum | Herbaspirillum sp. Sco-D20 | 0,21 | | 0,18 | | 0,00 | | 0,05 | | | | 0,457 | | 0,009 | | 0,169 | | 0,158 | |
| Proteobacteria | Alphaproteobacteria | Rhizobiales | | Methylobacteriaceae | Microvirga | Microvirga sp. SV2184P | 0,00 | | 0,06 | | 0,25 | | 0,09 | | | | 0,893 | | 0,671 | | 0,901 | | 0,526 | |
| Actinobacteria | Actinobacteria | Actinomycetales | | Micromonosporaceae | Actinoplanes | Actinoplanes sp. RI44-Va104 | 0,11 | | 0,32 | | 0,00 | | 0,00 | | | | 0,701 | | 0,072 | | 0,467 | | 0,424 | |
| Actinobacteria | Actinobacteria | Actinomycetales | | Microbacteriaceae | Agromyces | glacial ice bacterium G500K-1 | 0,00 | | 0,00 | | 0,27 | | 0,12 | | | | 0,240 | | 0,020 | | 0,044 | | 0,056 | |
| Actinobacteria | Actinobacteria | Acidimicrobiales | | Iamiaceae | Iamia | Iamia majanohamensis (T) | 0,10 | | 0,04 | | 0,16 | | 0,10 | | | | 0,381 | | 0,093 | | 0,901 | | 0,074 | |
| Actinobacteria | Actinobacteria | Actinomycetales | | Nocardioidaceae | Aeromicrobium | Aeromicrobium sp. ZS218-2 | 0,00 | | 0,00 | | 0,30 | | 0,07 | | | | 1,000 | | 0,031 | | 0,125 | | 0,116 | |
| Proteobacteria | Alphaproteobacteria | Rhizobiales | | Methylobacteriaceae | Methylobacterium | Methylobacterium sp. MG-2011-64-DP | 0,05 | | 0,25 | | 0,00 | | 0,13 | | | | 0,701 | | 0,072 | | 1,000 | | 0,007 | |
| Acidobacteria | Acidobacteria_Gp1 | Candidatus_Koribacter | | Candidatus_Koribacter | Candidatus_Koribacter | Candidatus Koribacter versatilis Ellin345 | 0,16 | | 0,21 | | 0,01 | | 0,02 | | | | 0,901 | | 0,243 | | 1,000 | | 1,000 | |
| Proteobacteria | Deltaproteobacteria | Myxococcales | | Kofleriaceae | Kofleria | Kofleria sp. DSM 53797 | 0,10 | | 0,06 | | 0,12 | | 0,10 | | | | 0,157 | | 0,387 | | 0,459 | | 0,248 | |
| Actinobacteria | Actinobacteria | Actinomycetales | | Nocardioidaceae | Nocardioides | Nocardioides sp. MSL 18 | 0,02 | | 0,01 | | 0,20 | | 0,15 | | | | 0,607 | | 0,293 | | 0,262 | | 0,530 | |
| Actinobacteria | Actinobacteria | Actinomycetales | | Micromonosporaceae | Actinoplanes | Micromonosporaceae bacterium 231729 | 0,05 | | 0,23 | | 0,08 | | 0,02 | | | | 0,268 | | 0,916 | | 0,171 | | 0,242 | |
| Actinobacteria | Actinobacteria | Actinomycetales | | Nocardioidaceae | Marmoricola | Kribbella sp. enrichment culture clone VanCtr42 | 0,03 | | 0,04 | | 0,16 | | 0,15 | | | | 0,530 | | 0,047 | | 0,385 | | 0,917 | |
| Actinobacteria | Actinobacteria | Actinomycetales | | Nakamurellaceae | Nakamurella | Nakamurella sp. 1153-i1wt | 0,00 | | 0,04 | | 0,20 | | 0,13 | | | | 0,602 | | 0,071 | | 0,381 | | 0,462 | |
| Gemmatimonadetes | Gemmatimonadetes | Gemmatimonadales | | Gemmatimonadaceae | Gemmatimonas | Gemmatimonadetes bacterium LP81 | 0,05 | | 0,07 | | 0,09 | | 0,16 | | | | 1,000 | | 0,222 | | 0,176 | | 0,599 | |
| Actinobacteria | Actinobacteria | Actinomycetales | | Pseudonocardiaceae | Actinophytocola | Actinophytocola oryzae (T) | 0,00 | | 0,00 | | 0,05 | | 0,35 | | | | 1,000 | | 0,022 | | 0,125 | | 0,451 | |
| Actinobacteria | Actinobacteria | Acidimicrobiales | | Acidimicrobiaceae | Ilumatobacter | Iamia sp. T2-YC6790 | 0,12 | | 0,05 | | 0,11 | | 0,07 | | | | 0,323 | | 0,458 | | 0,804 | | 0,020 | |
| Proteobacteria | Deltaproteobacteria | Myxococcales | | Polyangiaceae | Byssovorax | Byssovorax cruenta (T) | 0,08 | | 0,05 | | 0,13 | | 0,10 | | | | 0,532 | | 0,206 | | 0,459 | | 0,203 | |
| Chloroflexi | unclassified | unclassified | | unclassified | unclassified | unclassified | 0,10 | | 0,21 | | 0,00 | | 0,05 | | | | 0,171 | | 0,024 | | 0,268 | | 0,434 | |
| Proteobacteria | Gammaproteobacteria | Xanthomonadales | | Sinobacteraceae | Steroidobacter | bacterium D29 | 0,02 | | 0,02 | | 0,14 | | 0,18 | | | | 1,000 | | 0,343 | | 0,260 | | 1,000 | |
| Actinobacteria | Actinobacteria | Actinomycetales | | Intrasporangiaceae | Tetrasphaera | Tetrasphaera japonica (T) | 0,20 | | 0,11 | | 0,03 | | 0,00 | | | | 0,623 | | 0,140 | | 0,140 | | 0,130 | |
| Proteobacteria | Alphaproteobacteria | Caulobacterales | | Caulobacteraceae | unclassified | unclassified | 0,10 | | 0,10 | | 0,08 | | 0,06 | | | | 0,711 | | 0,399 | | 0,453 | | 0,334 | |
| Proteobacteria | Deltaproteobacteria | Myxococcales | | Polyangiaceae | Sorangium | Sorangium cellulosum | 0,03 | | 0,03 | | 0,16 | | 0,11 | | | | 0,797 | | 0,012 | | 0,077 | | 0,011 | |
| Acidobacteria | Acidobacteria_Gp3 | Gp3 | | Gp3 | Gp3 | Acidobacteria bacterium SCGC AAA204-D14 | 0,18 | | 0,16 | | 0,00 | | 0,00 | | | | 1,000 | | 0,264 | | 0,893 | | 0,723 | |
| Actinobacteria | Actinobacteria | Actinomycetales | | Pseudonocardiaceae | Amycolatopsis | Amycolatopsis mediterranei S699 | 0,17 | | 0,17 | | 0,00 | | 0,00 | | | | 0,902 | | 0,106 | | 0,247 | | 1,000 | |
| Bacteroidetes | unclassified | unclassified | | unclassified | unclassified | unclassified | 0,00 | | 0,05 | | 0,16 | | 0,12 | | | | 0,787 | | 0,071 | | 0,383 | | 0,421 | |
| Bacteroidetes | Sphingobacteriia | Sphingobacteriales | | Chitinophagaceae | Segetibacter | Segetibacter aerophilus | 0,02 | | 0,10 | | 0,03 | | 0,22 | | | | 0,797 | | 0,831 | | 0,453 | | 0,525 | |
| Gemmatimonadetes | Gemmatimonadetes | Gemmatimonadales | | Gemmatimonadaceae | Gemmatimonas | Gemmatimonadetes bacterium enrichment culture clone phylotype P10 | 0,03 | | 0,00 | | 0,10 | | 0,21 | | | | 0,306 | | 0,753 | | 0,201 | | 0,753 | |
| Proteobacteria | Alphaproteobacteria | Caulobacterales | | Caulobacteraceae | Caulobacter | Caulobacter vibrioides | 0,06 | | 0,07 | | 0,13 | | 0,06 | | | | 1,000 | | 0,015 | | 1,000 | | 0,021 | |
| Proteobacteria | Gammaproteobacteria | Xanthomonadales | | Sinobacteraceae | Steroidobacter | Steroidobacter sp. WWH78 | 0,00 | | 0,02 | | 0,17 | | 0,13 | | | | 0,893 | | 0,033 | | 0,133 | | 0,093 | |
| Proteobacteria | Alphaproteobacteria | Rhizobiales | | Bradyrhizobiaceae | Bradyrhizobium | Bradyrhizobium valentinum | 0,00 | | 0,00 | | 0,20 | | 0,10 | | | | 0,240 | | 0,057 | | 0,044 | | 0,528 | |
| Actinobacteria | Actinobacteria | Actinomycetales | | Nocardioidaceae | Nocardioides | actinobacterium GWS-BW-H259 | 0,02 | | 0,00 | | 0,17 | | 0,12 | | | | 0,687 | | 0,401 | | 0,687 | | 0,171 | |
| Bacteroidetes | Bacteroidetes_incertae_sedis | Ohtaekwangia | | Ohtaekwangia | Ohtaekwangia | unidentified bacterium | 0,03 | | 0,04 | | 0,17 | | 0,05 | | | | 0,617 | | 0,046 | | 0,459 | | 0,036 | |
| Actinobacteria | Actinobacteria | Solirubrobacterales | | Conexibacteraceae | Conexibacter | Conexibacter sp. L1948 | 0,06 | | 0,03 | | 0,17 | | 0,03 | | | | 0,157 | | 0,206 | | 1,000 | | 0,046 | |
| Acidobacteria | Acidobacteria_Gp3 | Gp3 | | Gp3 | Gp3 | Actinomycetales | 0,13 | | 0,07 | | 0,00 | | 0,12 | | | | 0,617 | | 0,160 | | 0,617 | | 0,160 | |
| Acidobacteria | Acidobacteria_Gp3 | Gp3 | | Gp3 | Gp3 | uncultivated soil bacterium clone C002 | 0,05 | | 0,06 | | 0,16 | | 0,02 | | | | 1,000 | | 0,142 | | 0,617 | | 0,141 | |
| Proteobacteria | Alphaproteobacteria | Rhizobiales | | Xanthobacteraceae | Pseudolabrys | methanogenic prokaryote enrichment culture B19_111 | 0,12 | | 0,06 | | 0,06 | | 0,06 | | | | 0,286 | | 0,401 | | 1,000 | | 0,396 | |
| Actinobacteria | Actinobacteria | Actinomycetales | | Nakamurellaceae | Nakamurella | Nakamurella multipartita DSM 44233 | 0,02 | | 0,00 | | 0,13 | | 0,15 | | | | 0,245 | | 0,015 | | 0,073 | | 0,753 | |
| Chloroflexi | Ktedonobacteria | Ktedonobacterales | | unclassified | unclassified | unclassified | 0,19 | | 0,10 | | 0,00 | | 0,00 | | | | 0,701 | | 0,072 | | 0,687 | | 0,180 | |
| Actinobacteria | Actinobacteria | Actinomycetales | | Nocardioidaceae | Nocardioides | Nocardioides sp. 02SU6 | 0,12 | | 0,05 | | 0,07 | | 0,05 | | | | 0,108 | | 0,746 | | 1,000 | | 0,193 | |
| Actinobacteria | Actinobacteria | Acidimicrobiales | | Acidimicrobineae_incertae_sedis | Aciditerrimonas | Aciditerrimonas ferrireducens (T) | 0,08 | | 0,05 | | 0,05 | | 0,12 | | | | 0,459 | | 0,914 | | 0,266 | | 0,665 | |
| Proteobacteria | Alphaproteobacteria | Rhodospirillales | | Rhodospirillaceae | Skermanella | Azospirillum sp. I54 | 0,03 | | 0,00 | | 0,07 | | 0,20 | | | | 0,348 | | 0,599 | | 0,073 | | 0,548 | |
| Firmicutes | Bacilli | Bacillales | | unclassified | unclassified | unclassified | 0,05 | | 0,08 | | 0,07 | | 0,10 | | | | 0,805 | | 0,338 | | 0,902 | | 1,000 | |
| Actinobacteria | Actinobacteria | Actinomycetales | | Nocardioidaceae | Pimelobacter | Nocardioides aromaticivorans | 0,10 | | 0,05 | | 0,10 | | 0,02 | | | | 0,321 | | 1,000 | | 0,900 | | 0,287 | |
| Actinobacteria | Actinobacteria | Actinomycetales | | Pseudonocardiaceae | Actinomycetospora | bacterium Ellin5115 | 0,00 | | 0,00 | | 0,18 | | 0,09 | | | | 1,000 | | 0,091 | | 0,441 | | 0,142 | |
| Actinobacteria | Actinobacteria | Actinomycetales | | Nocardioidaceae | Marmoricola | Marmoricola bigeumensis (T) | 0,07 | | 0,08 | | 0,08 | | 0,05 | | | | 1,000 | | 0,525 | | 0,701 | | 0,092 | |
| Actinobacteria | Actinobacteria | Solirubrobacterales | | Solirubrobacteraceae | Solirubrobacter | Solirubrobacter sp. L1977 | 0,02 | | 0,02 | | 0,13 | | 0,12 | | | | 0,418 | | 0,074 | | 0,526 | | 0,462 | |
| Actinobacteria | Actinobacteria | Gaiellales | | Gaiellaceae | Gaiella | grassland soil clone saf2_202 | 0,02 | | 0,00 | | 0,17 | | 0,08 | | | | 0,348 | | 0,094 | | 0,125 | | 0,249 | |
| Actinobacteria | Actinobacteria | Actinomycetales | | Intrasporangiaceae | Terrabacter | Terrabacter lapilli (T) | 0,16 | | 0,10 | | 0,00 | | 0,02 | | | | 0,898 | | 0,072 | | 0,898 | | 0,072 | |
| Proteobacteria | Alphaproteobacteria | Caulobacterales | | Caulobacteraceae | Phenylobacterium | Phenylobacterium immobile | 0,03 | | 0,03 | | 0,09 | | 0,12 | | | | 0,453 | | 0,340 | | 0,048 | | 1,000 | |
| Proteobacteria | Alphaproteobacteria | Rhizobiales | | Hyphomicrobiaceae | unclassified | unclassified | 0,00 | | 0,07 | | 0,14 | | 0,06 | | | | 0,131 | | 0,025 | | 1,000 | | 0,242 | |
| Actinobacteria | Actinobacteria | Actinomycetales | | Micrococcaceae | Arthrobacter | Arthrobacter sp. L13 | 0,02 | | 0,25 | | 0,00 | | 0,02 | | | | 0,898 | | 0,072 | | 1,000 | | 0,071 | |
| Proteobacteria | Alphaproteobacteria | Rhizobiales | | Bradyrhizobiaceae | Bradyrhizobium | Bradyrhizobium sp. NBRC 101128 | 0,03 | | 0,00 | | 0,09 | | 0,15 | | | | 0,093 | | 0,073 | | 0,024 | | 0,399 | |
| Actinobacteria | Actinobacteria | Actinomycetales | | Micromonosporaceae | Virgisporangium | Virgisporangium aurantiacum (T) | 0,03 | | 0,00 | | 0,12 | | 0,12 | | | | 0,687 | | 0,035 | | 0,443 | | 0,401 | |
| Gemmatimonadetes | Gemmatimonadetes | Gemmatimonadales | | Gemmatimonadaceae | Gemmatimonas | bacterium enrichment culture clone R1492-14 | 0,01 | | 0,00 | | 0,14 | | 0,10 | | | | 0,338 | | 0,199 | | 0,073 | | 0,463 | |
| Acidobacteria | Acidobacteria_Gp1 | Gp1 | | Gp1 | Gp1 | Acidobacteriaceae bacterium A2-1c | 0,14 | | 0,12 | | 0,00 | | 0,00 | | | | 0,898 | | 0,158 | | 0,467 | | 1,000 | |
| Actinobacteria | Actinobacteria | Actinomycetales | | Pseudonocardiaceae | Amycolatopsis | Amycolatopsis sp. GDS | 0,12 | | 0,15 | | 0,00 | | 0,00 | | | | 1,000 | | 0,066 | | 0,123 | | 1,000 | |
| Bacteroidetes | Sphingobacteriia | Sphingobacteriales | | Chitinophagaceae | Flavisolibacter | Flavisolibacter sp. Gsoil 636 | 0,08 | | 0,04 | | 0,08 | | 0,06 | | | | 0,321 | | 1,000 | | 1,000 | | 0,344 | |
| Bacteroidetes | Sphingobacteriia | Sphingobacteriales | | Chitinophagaceae | Flavisolibacter | bacterium enrichment culture clone SC-2_14 | 0,07 | | 0,10 | | 0,05 | | 0,04 | | | | 0,533 | | 0,456 | | 0,323 | | 0,672 | |
| Proteobacteria | Alphaproteobacteria | Rhizobiales | | Bradyrhizobiaceae | Bradyrhizobium | Bradyrhizobium sp. VUPME29 | 0,03 | | 0,00 | | 0,16 | | 0,05 | | | | 0,281 | | 0,027 | | 0,024 | | 0,092 | |
| Proteobacteria | Gammaproteobacteria | Pseudomonadales | | Pseudomonadaceae | Rhizobacter | Rhizobacter fulvus | 0,06 | | 0,02 | | 0,03 | | 0,15 | | | | 0,797 | | 0,916 | | 0,063 | | 0,165 | |
| Bacteroidetes | Sphingobacteriia | Sphingobacteriales | | Chitinophagaceae | Flavisolibacter | Flavisolibacter sp. enrichment culture clone 02SUJ3 | 0,08 | | 0,11 | | 0,03 | | 0,03 | | | | 1,000 | | 0,596 | | 0,215 | | 0,338 | |
| Actinobacteria | Actinobacteria | Actinomycetales | | Geodermatophilaceae | Geodermatophilus | Geodermatophilus | 0,13 | | 0,02 | | 0,06 | | 0,03 | | | | 0,532 | | 0,461 | | 0,900 | | 0,461 | |
| Actinobacteria | Actinobacteria | Actinomycetales | | Microbacteriaceae | Agromyces | Agromyces ramosus | 0,02 | | 0,00 | | 0,13 | | 0,09 | | | | 0,441 | | 0,246 | | 0,441 | | 0,203 | |
| Actinobacteria | Actinobacteria | Solirubrobacterales | | Conexibacteraceae | Conexibacter | bacterium Ellin5025 | 0,09 | | 0,13 | | 0,02 | | 0,02 | | | | 0,730 | | 0,020 | | 0,375 | | 0,507 | |
| Nitrospirae | Nitrospira | Nitrospirales | | Nitrospiraceae | Nitrospira | Nitrospira japonica | 0,08 | | 0,01 | | 0,09 | | 0,06 | | | | 0,163 | | 0,249 | | 0,033 | | 0,752 | |
| Actinobacteria | Actinobacteria | Actinomycetales | | Thermomonosporaceae | unclassified | unclassified | 0,05 | | 0,11 | | 0,00 | | 0,10 | | | | 0,623 | | 0,230 | | 0,901 | | 0,065 | |
| Proteobacteria | Alphaproteobacteria | Rhizobiales | | Bradyrhizobiaceae | Bradyrhizobium | Bradyrhizobium group bacterium Ellin127 | 0,03 | | 0,00 | | 0,11 | | 0,10 | | | | 0,281 | | 0,092 | | 0,072 | | 0,092 | |
| Actinobacteria | Actinobacteria | Actinomycetales | | Pseudonocardiaceae | Pseudonocardia | Pseudonocardiaceae | 0,00 | | 0,17 | | 0,05 | | 0,03 | | | | 0,140 | | 0,119 | | 0,262 | | 0,594 | |
| Proteobacteria | Gammaproteobacteria | Xanthomonadales | | Sinobacteraceae | Steroidobacter | Pseudomonas sp. VT1B | 0,03 | | 0,00 | | 0,08 | | 0,15 | | | | 0,687 | | 0,055 | | 0,441 | | 0,597 | |
| Proteobacteria | Alphaproteobacteria | Rhodospirillales | | Rhodospirillaceae | Dongia | bacterium Ellin314 | 0,12 | | 0,00 | | 0,08 | | 0,03 | | | | 0,201 | | 0,599 | | 0,441 | | 0,034 | |
| Actinobacteria | Actinobacteria | Actinomycetales | | Micrococcaceae | Arthrobacter | Arthrobacter sp. CFM 20 | 0,18 | | 0,06 | | 0,00 | | 0,00 | | | | 0,902 | | 0,025 | | 0,042 | | NA | |
| Actinobacteria | Actinobacteria | Actinomycetales | | Intrasporangiaceae | Terrabacter | Terrabacter terrae (T) | 0,14 | | 0,09 | | 0,00 | | 0,00 | | | | 0,709 | | 0,158 | | 0,042 | | 0,424 | |
| Actinobacteria | Actinobacteria | Actinomycetales | | Geodermatophilaceae | Geodermatophilus | Geodermatophilus saharensis (T) | 0,08 | | 0,13 | | 0,01 | | 0,02 | | | | 0,902 | | 0,199 | | 0,260 | | 1,000 | |
| Actinobacteria | Actinobacteria | Actinomycetales | | Micrococcaceae | Arthrobacter | Arthrobacter sp. HS4-1 | 0,19 | | 0,03 | | 0,00 | | 0,00 | | | | 0,522 | | 0,072 | | 0,467 | | 0,424 | |
| Proteobacteria | Gammaproteobacteria | Xanthomonadales | | Xanthomonadaceae | Rudaea | unidentified bacterium | 0,15 | | 0,07 | | 0,00 | | 0,00 | | | | 0,901 | | 0,072 | | 0,609 | | 0,180 | |
| Proteobacteria | Alphaproteobacteria | Caulobacterales | | Caulobacteraceae | Phenylobacterium | Phenylobacterium sp. P-28 | 0,05 | | 0,03 | | 0,06 | | 0,08 | | | | 0,610 | | 0,831 | | 0,321 | | 0,831 | |
| Actinobacteria | Actinobacteria | Solirubrobacterales | | Solirubrobacteraceae | Solirubrobacter | bacterium Ellin404 | 0,04 | | 0,00 | | 0,11 | | 0,05 | | | | 0,156 | | 0,036 | | 0,116 | | 0,056 | |
| Proteobacteria | Alphaproteobacteria | Rhizobiales | | Hyphomicrobiaceae | Pedomicrobium | Pedomicrobium manganicum (T) | 0,00 | | 0,00 | | 0,07 | | 0,17 | | | | 0,240 | | 0,014 | | 0,107 | | 0,673 | |
| Gemmatimonadetes | Gemmatimonadetes | Gemmatimonadales | | Gemmatimonadaceae | Gemmatimonas | Gemmatimonas sp. enrichment culture clone AOCRB-EC-6 | 0,03 | | 0,08 | | 0,05 | | 0,06 | | | | 0,900 | | 0,916 | | 1,000 | | 0,828 | |
| Bacteroidetes | Sphingobacteriia | Sphingobacteriales | | Chitinophagaceae | Terrimonas | unidentified bacterium | 0,02 | | 0,01 | | 0,10 | | 0,08 | | | | 0,699 | | 0,045 | | 0,133 | | 0,243 | |
| Actinobacteria | Actinobacteria | Actinomycetales | | Nocardioidaceae | Nocardioides | bacterium Ellin6014 | 0,10 | | 0,08 | | 0,03 | | 0,00 | | | | 0,900 | | 0,287 | | 0,787 | | 0,655 | |
| Bacteroidetes | Flavobacteriia | Flavobacteriales | | Flavobacteriaceae | unclassified | unclassified | 0,03 | | 0,08 | | 0,07 | | 0,04 | | | | 0,700 | | 0,338 | | 1,000 | | 0,671 | |
| Actinobacteria | Actinobacteria | Actinomycetales | | Nocardioidaceae | Pimelobacter | Nocardioides sp. 2145C | 0,05 | | 0,04 | | 0,08 | | 0,05 | | | | 0,900 | | 0,160 | | 0,797 | | 0,246 | |
| Proteobacteria | Alphaproteobacteria | Rhizobiales | | Bradyrhizobiaceae | Bradyrhizobium | bacterium Ellin6538 | 0,03 | | 0,12 | | 0,03 | | 0,03 | | | | 0,701 | | 0,830 | | 0,900 | | 1,000 | |
| Actinobacteria | Actinobacteria | Actinomycetales | | Micrococcaceae | Arthrobacter | soil bacterium 14V-08 | 0,17 | | 0,04 | | 0,00 | | 0,00 | | | | 0,709 | | 0,025 | | 0,500 | | 0,177 | |
| Actinobacteria | Actinobacteria | Actinomycetales | | Micromonosporaceae | Actinoplanes | Actinoplanes cyaneus (T) | 0,03 | | 0,16 | | 0,02 | | 0,00 | | | | 0,264 | | 0,395 | | 0,123 | | 0,822 | |
| Proteobacteria | Betaproteobacteria | Rhodocyclales | | Rhodocyclaceae | unclassified | unclassified | 0,06 | | 0,00 | | 0,07 | | 0,08 | | | | 0,109 | | 0,523 | | 0,044 | | 1,000 | |
| Actinobacteria | Actinobacteria | Actinomycetales | | Micrococcaceae | Arthrobacter | Arthrobacter sp. JCM 1339 | 0,10 | | 0,11 | | 0,00 | | 0,00 | | | | 1,000 | | 0,072 | | 0,081 | | 0,424 | |
| Actinobacteria | Actinobacteria | Actinomycetales | | Micrococcaceae | Arthrobacter | Arthrobacter sp. EP_L_35 | 0,15 | | 0,05 | | 0,00 | | 0,00 | | | | 0,537 | | 0,067 | | 0,606 | | 0,724 | |
| Bacteroidetes | Sphingobacteriia | Sphingobacteriales | | Chitinophagaceae | Flavisolibacter | bacterium enrichment culture clone SC-2_38 | 0,07 | | 0,04 | | 0,04 | | 0,06 | | | | 0,899 | | 0,459 | | 1,000 | | 0,452 | |
| Chloroflexi | Chloroflexia | Chloroflexales | | Chloroflexaceae | unclassified | unclassified | 0,10 | | 0,11 | | 0,00 | | 0,00 | | | | 0,902 | | 0,025 | | 0,247 | | 0,180 | |
| Proteobacteria | Alphaproteobacteria | Rhizobiales | | Bradyrhizobiaceae | Balneimonas | Microvirga flocculans (T) | 0,02 | | 0,01 | | 0,16 | | 0,00 | | | | 0,607 | | 0,115 | | 0,893 | | 0,057 | |
| Actinobacteria | Actinobacteria | Actinomycetales | | Cryptosporangiaceae | Cryptosporangium | Cryptosporangium minutisporangium (T) | 0,08 | | 0,00 | | 0,08 | | 0,03 | | | | 0,44 | | 0,833 | | 0,439 | | 0,165 | |
| Actinobacteria | Actinobacteria | | Actinomycetales | Intrasporangiaceae | Terrabacter | Terrabacter sp. A2-62 | 0,10 | | | 0,11 | | 0,00 | | 0,00 | | | | 0,701 | | 0,158 | | 0,308 | | 1,000 |
