# Supplementary figures and images for: The Rhizosphere Bacterial Microbiota of Vitis vinifera cv. Pinot Noir in an Integrated Pest Management Vineyard
Source: Front Microbiol. 2017 Aug 14;8:1528. doi: 10.3389/fmicb.2017.01528 (PMC5557794; doi:10.3389/fmicb.2017.01528)

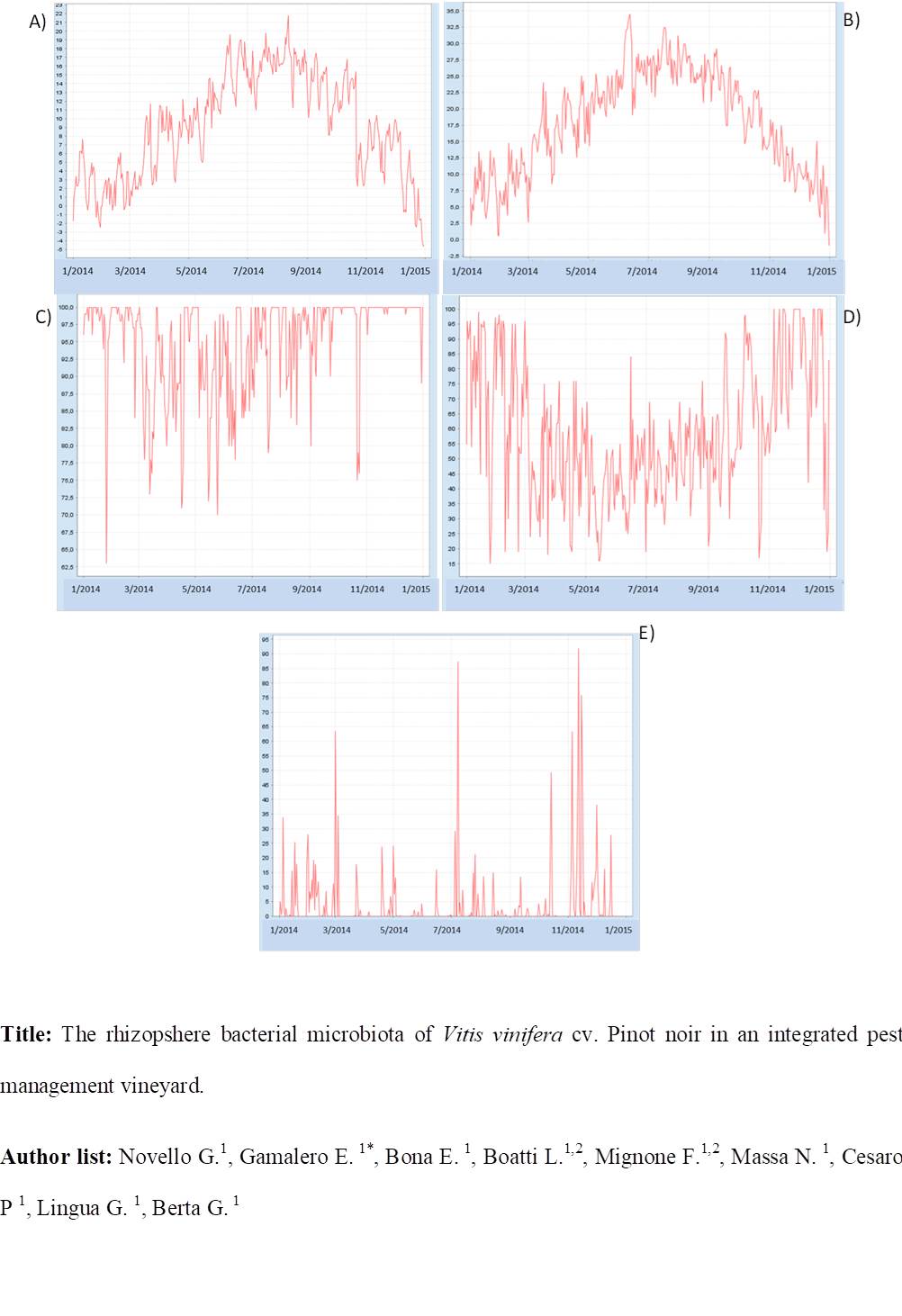

Supplement: Supplementary file 2 [file Image_1.JPEG]
